# Predicting bloodstream infection by plasma cell-free metagenomic sequencing: a prospective cohort study

**DOI:** 10.1016/j.lanmic.2025.101312

**Published:** 2026-03-02

**Authors:** Joshua Wolf, Kathryn P Goggin, Yuki Inaba, Kim J Allison, Asim A Ahmed, Gabriela Maron, Jose Ferrolino, Lauren Lazure, Christina Kohler, Abigail Brenner, Yilun Sun, Li Tang, Veronica Gonzalez-Pena, Jeffrey E Rubnitz, Charles Gawad, Elisa B Margolis, Paul Thomas

**Affiliations:** **Department of Infectious Diseases, St Jude Children’s Research Hospital, Memphis, TN, USA** (Prof J Wolf MBBS PhD, K P Goggin MD, K J Allison BSN, G Maron MD MS, J Ferrolino MD, L Lazure BS, C Kohler MS, A Brenner MD,E B Margolis MD PhD);**Department of Pediatrics, University of Tennessee Health Science Center, Memphis, TN, USA** (Prof J Wolf, Y Inaba MS); **Department of Pediatrics, Boston Children’s Hospital, Boston, MA, USA** (A A Ahmed MD); **Department of Biostatistics, St Jude Children’s Research Hospital, Memphis, TN, USA** (Y Sun PhD, Prof L Tang PhD); **Department of Pathology, St Jude Children’s Research Hospital, Memphis, TN, USA** (V Gonzalez-Pena PhD, C Gawad MD PhD); **Department of Oncology, St Jude Children’s Research Hospital, Memphis, TN, USA** (J E Rubnitz MD PhD, C Gawad)**; Department of Pediatrics, Stanford University, Stanford, CA, USA** (C Gawad); **ementDepartment of Host–Microbe Interactions, St Jude Children’s Research Hospital, Memphis, TN, USA** (E B Margolis, Prof P Thomas PhD)

## Abstract

**Background:**

Patients receiving myelosuppressive chemotherapy or haematopoietic cell transplantation are at high risk for life-threatening bloodstream infections. A novel pre-emptive treatment paradigm guided by pathogen detection before symptoms appear might reduce this risk, but no validated screening test is available. This study evaluated the sensitivity and specificity of plasma microbial cell-free DNA metagenomic sequencing (mcfDNA-Seq) for predicting bloodstream infections in children and adolescents receiving therapy for high-risk leukaemia.

**Methods:**

In this prospective cohort study, between Aug 9, 2017, and Feb 28, 2022, leftover clinical plasma samples were prospectively collected up to once per day from patients who were younger than 25 years, receiving care for leukaemia at St Jude Children’s Research Hospital (Memphis, TN, USA), and at high risk for life-threatening bloodstream infections. mcfDNA-Seq was used to identify pathogen DNA in blood samples obtained during the 7 days before to 1 day after bloodstream infection onset, and in control samples from the same population in the absence of fever or infection. The testing laboratory was masked to sample status. Primary outcomes were predictive sensitivity of mcfDNA-Seq for detecting the expected bloodstream infection pathogen during the 3 days preceding the day of bloodstream infection onset, with a prespecified favourable sensitivity of 50%, and predictive specificity of mcfDNA-Seq in control samples. Exploratory analyses comprised assessing sensitivity and specificity restricted to bacteria or common bloodstream infection pathogens, and after applying a data-derived DNA fragment concentration cutoff; estimating the predictive sensitivity on each of the 7 days before bloodstream infection onset; identifying clinical characteristics that affected predictive sensitivity or specificity; and examining the clinical relevance of additional organisms identified by mcfDNA-Seq during bloodstream infection episodes. Diagnostic sensitivity was also assessed on samples collected on the day of, or day after, diagnosis of bloodstream infection. This study is registered with ClinicalTrials.gov, NCT03226158.

**Findings:**

94 evaluable bloodstream infections occurred in 60 (38%) of 158 enrolled participants; 19 episodes were previously described in the pilot phase of this study. The predictive sensitivity of mcfDNA-Seq was 51·9% (95% CI 40·5–63·1) for all bloodstream infection episodes, 53·8% (42·2–65·2) for bacterial infection only, and 51·9% (40·5–63·1) when applying a DNA fragment concentration cutoff of 140 molecules per μL. Sensitivity was lowest at day −7 and increased daily until the day of diagnosis. Diagnostic sensitivity was 81·3% (95% CI 71·0–89·1) for all bloodstream infection episodes and 83·1% (72·9–90·7) for bacterial infections only. Predictive specificity was 82·7% (95% CI 76·0–88·2), but improved to 88·9% (83·0–93·3) for common bloodstream infection pathogens, and to 93·8% (88·9–97·0) when also applying the DNA fragment concentration cutoff. Predictive sensitivity was higher in participants with acute lymphoblastic leukaemia (adjusted odds ratio [aOR] 11·1 [1·7–74·2] *vs* those with acute myeloid leukaemia), and it was lower in polymicrobial infections (aOR 0·0 [0·0–0·2] *vs* monomicrobial Grampositive infections). Clinical false-positive results were positively associated with gastrointestinal disturbance alone (p=0·037) or combined with recent administration of high-dose cytarabine (p=0·012). Additional organisms identified by mcfDNA-Seq that were not identified by blood culture were less likely than expected organisms to have an increasing DNA concentration during the days preceding bloodstream infection diagnosis.

**Interpretation:**

mcfDNA-Seq can detect causative pathogens before the onset of some bloodstream infection episodes in profoundly immunocompromised patients. Predictive specificity might be improved by restricting results to a subgroup of relevant organisms, excluding patients with high risk of false-positive results, or applying a higher concentration cutoff. Clinical trials are needed to evaluate mcfDNA-Seq-guided pre-emptive therapy for preventing life-threatening bloodstream infections in patients with high risk.

## Introduction

Children and adolescents with leukaemia receiving chemotherapy or undergoing haematopoietic cell transplantation (HCT) are at extreme risk for life-threatening bloodstream infections and sepsis.^1–5^ Potential impacts of these complications include delay or discontinuation of effective chemotherapy, increased risk of long-term sequelae such as osteonecrosis (odds are increased by 88% by bloodstream infections during treatment for paediatric acute lymphoblastic leukaemia) and neurocognitive dysfunction (hazard is increased by 86% by severe sepsis during treatment for paediatric leukaemia), and risk of infection-related mortality, with more than 25% of all deaths being attributed to infection.^1–8^ Although risk- stratified antibacterial prophylaxis reduces bloodstream infection risk by about 50%, serious harms, such as increasing antibiotic resistance and breakthrough infection, could restrict the long-term usefulness of this approach.^3,9–13^ Therefore, a novel paradigm of predicting specific bloodstream infection events before their onset, with the ability to provide targeted pre-emptive antimicrobial therapy, would be a breakthrough for these patients.

Cell-free DNA (cfDNA) refers to short fragments of DNA, not contained within cells, that can be detected in body fluids such as blood or cerebrospinal fluid. cfDNA has shown promise for diagnosing and monitoring cancer, infectious diseases, and prenatal genetic abnormalities.^14,15^ Microbial cfDNA metagenomic sequencing (mcfDNA-Seq) directly from blood is a rapid, clinically available laboratory test that has shown promise for diagnosing infection, including in immunocompromised hosts.^15^ However, the predictive value of mcfDNA-Seq before the onset of infection and its specificity in immunocompromised patients without infection are not well defined. A small observational study evaluating mcfDNA-Seq prediction of bloodstream infection in adults in the intensive care unit estimated the predictive sensitivity as 87·5%.^16^ A larger study evaluating the prediction of invasive pulmonary fungal infections in adult patients undergoing HCT estimated the predictive sensitivity as up to 37·5% in the week before diagnosis.^17^ In the pilot phase of the present study, comprising 19 bloodstream infection episodes, DNA from the causative pathogen could be detected by mcfDNA-Seq before the onset of clinically detectable bloodstream infections in most cases.^18^ However, immunocompromised patients might be more likely than others to have circulating pathogen DNA without clinical infection as a result of mucosal damage resulting from chemotherapy or graft-versus-host disease (GVHD), leading to decreased specificity.^15,19^

The aims of this work were to determine whether mcfDNA-Seq was a candidate tool for predictive testing for bloodstream infections, to develop an evidence-based testing paradigm, and to identify populations that would benefit most from this novel strategy.

## Methods

### Study design and participants

This prospective observational cohort study was undertaken at St Jude Children’s Research Hospital between Aug 9, 2017, and Feb 28, 2022.^18^ The study was approved by the St Jude Institutional Review Board (approval number Pro00007383). All participants or their representatives provided written informed consent for research use of leftover clinical samples, and separately for prospective data abstraction from the electronic medical record until the second requirement was waived by the institutional review board on July 31, 2020, because of institutional COVID-19 pandemic procedures (according to US Department of Health and Human Services regulation 45 CFR 46.116d). The participants—individuals younger than 25 years with cancer who were at high risk for infection and were expected to receive care at St Jude Children’s Research Hospital for at least 7 days—were prospectively enrolled and remained on study until loss to follow-up, death, 30 days after HCT, or resolution of gastrointestinal GVHD. Reporting of the study is divided into a previously reported pilot phase^18^ and a completion phase, with both new and consolidated data reported here as specified below. This study is registered with ClinicalTrials.gov, NCT03226158.

### Procedures

Leftover blood collected for clinical haematology testing was prospectively obtained, stored at 4° C until processed to plasma, then frozen at −80° C. Demographic, treatment, and infection data were collected from the electronic medical record and institutional databases and recorded in study-specific, restricted-access databases.

Bloodstream infection was defined according to the National Healthcare Safety Network Laboratory-Confirmed Bloodstream Infection classification, with onset being set at the date and time of collection of the first positive blood culture.^20^ Institutional practice is to collect blood cultures from all lumens of indwelling central venous catheters, and peripheral blood at clinician discretion, from patients with new-onset fever or clinical concern for infection (appendix p 2). If fever persists, blood cultures are collected daily for 2 days or with subsequent clinical deterioration.^21^

The a priori-defined predictive period was from day −3 to day −1 before bloodstream infection onset, and prediction up to 7 days before onset was also explored; predictive sensitivity was defined as the detection of all culture- confirmed bloodstream infection organisms by mcfDNA- Seq during the predictive period. The diagnostic period was from day 0 (the date of diagnosis) to day 1; diagnostic sensitivity was defined as the detection of all culture- confirmed bloodstream infection organisms by mcfDNA- Seq during the diagnostic period. The primary analysis of predictive and diagnostic sensitivity included all bloodstream infection organisms meeting National Healthcare Safety Network criteria;^20^ additional sensitivity analyses restricted to bacteria or to common bloodstream infection pathogens, and after applying a data-derived molecules-per-μL cutoff were also done.

Control episode samples were collected from participants who had no fever or infection identified within 7 calendar days before or after sample collection. Common bloodstream infection pathogens were genera accounting for more than 1% of central line-associated bloodstream infection episodes in children with cancer according to the Children’s Hospital Association Childhood Cancer & Blood Disorders Network Bloodstream Infections database, compiled between Aug 1, 2013, and Sept 30, 2015, as previously described (appendix p 4).^18,22^ Specificity was defined as the detection of no bacteria or yeast by mcfDNA-Seq in control samples.

Bloodstream infection-related samples (collected between 7 days before and 1 day after bloodstream infection onset), along with up to two control samples per episode from the same cohort, underwent mcfDNA-Seq in a Clinical Laboratory Improvement Amendments-accredited and College of American Pathologists-accredited laboratory (Karius, Redwood City, CA, USA), and the concentration of microbial DNA fragments for each organism identified using a proprietary reference-genome database was reported in molecules per μL of plasma (appendix pp 2–3). Testing was batched, and the laboratory was masked to sample status.

### Outcomes

The primary outcomes were predictive sensitivity of mcfDNA-Seq during the a priori-defined predictive period, and predictive specificity in the absence of fever or documented infection in the 7 days before or after sample collection. Additional exploratory outcomes were: predictive sensitivity on each of the 7 days before bloodstream infection onset; diagnostic sensitivity on the day of or day after diagnosis of bloodstream infection; predictive sensitivity and specificity restricted to bacterial bloodstream infections or common bloodstream infection pathogens, and after applying a post-hoc, data-derived molecules-per-μL cutoff; clinical characteristics that affected the predictive sensitivity or specificity; and the clinical relevance of additional organisms identified by mcfDNA-Seq during bloodstream infection episodes.

Statistical analysis

The study followed Simon’s optimal two-stage design, with a favourable sensitivity of 50% and a target sample size of 100 evaluable bloodstream infection episodes (appendix p 3). Data available at completion of the first stage (the pilot phase) have been reported previously;^18^ both new data from this completion phase and consolidated data are reported here as specified. In this phase, the aim was to estimate more precisely the sensitivity and specificity of mcfDNA-Seq for impending bloodstream infections, explore the clinical significance of mcfDNA-Seq-detected additional organisms, and identify populations at high risk for clinical false positives. Each bloodstream infection episode was treated as an independent event in analyses.

Sensitivity was evaluated for the predictive period (≤3 days before bloodstream infection onset), separately for each of the 7 days before bloodstream infection onset, and at diagnosis, using raw data or logical imputation for missing values (appendix p 3).^18^ Predictive sensitivity was defined as the proportion of evaluable bloodstream infection episodes with mcfDNA-Seq available for at least one predictive sample for which the organisms identified in blood culture were also detected by mcfDNA-Seq in at least one sample from the predictive period (including all organisms identified by culture in polymicrobial bloodstream infection episodes). Diagnostic sensitivity was defined as the proportion of evaluable bloodstream infection episodes with mcfDNA-Seq available during the diagnostic period for which the organisms identified in blood culture were also detected by mcfDNA-Seq in at least one sample from the diagnostic period (including all organisms identified by culture in polymicrobial bloodstream infection episodes). The primary analysis of predictive sensitivity included all bloodstream infection episodes, and exploratory analyses restricted to bacteria or to common bloodstream infection pathogens and after applying a data-derived molecules-per-μL cutoff were done.

Factors that might affect the predictive sensitivity of mcfDNA-Seq—including age, sample timing, leukaemia type, HCT or GVHD, haematological parameters, sepsis, time to blood culture positivity, and organism group—were evaluated in generalised linear mixed models, yielding adjusted odds ratios (aORs) for sensitivity with 95% CIs; variables with p values less than 0·05 in the univariate analysis were included in a multivariable analysis.

Predictive specificity was defined as the proportion of evaluable control samples in which no bacteria or yeast was detected by mcfDNA-Seq. The primary analysis for specificity included bacteria or yeasts only; parasites, moulds, and viruses were excluded a priori, and *Burkholderia cepacia* was excluded subsequently because of possible local sample contamination (appendix p 3).^23^ Additional analyses restricted to common bloodstream infection pathogens and after applying a data-derived molecules-per-μL cutoff that maximised the Youden index selected from a receiver operating characteristic curve analysis^24^ were also done. Variables associated with clinical false-positive detection of pathogens by mcfDNA-Seq in control samples were evaluated with Fisher’s exact test or the Wilcoxon–Mann–Whitney test as appropriate.

Because mcfDNA-Seq might detect bacteria present in low numbers or affected by antibiotics—leading to greater sensitivity than blood culture—or detect genetic material from organisms not causing disease—leading to lower specificity—the clinical significance of the additional organisms identified by mcfDNA-Seq at diagnosis of bloodstream infection episodes was assessed in exploratory analyses that estimated the proportion of diagnostic samples with any additional bacterial or fungal organisms detected above the data-derived cutoff, that were not treated with at least 3 days of antibiotics expected to be effective based on published data^25,26^ or local epidemiology (classified as potentially effective antimicrobial therapy), and that did not lead to a microbiologically documented infection within 7 days. Trajectory analysis using data visualisation was used to explore whether DNA from expected organisms was more likely than DNA from additional organisms to be increasing in the days preceding bloodstream infection onset.

All tests were two-sided, and statistical significance was set at a p value less than 0·05. Data analyses were done with R (version 4.3.1).

### Role of the funding source

The American Lebanese Syrian Associated Charities (Memphis, TN, USA) and the National Cancer Institute (Bethesda, MD, USA) had no role in design and conduct of the study; collection, management, analysis, and interpretation of data; preparation, review, or approval of the manuscript; or the decision to submit the manuscript for publication. Representatives of Karius were involved in sample processing and analysis, data management, and reviewing the manuscript, but had no role in the preparation, writing, or approval of the manuscript or in the decision to submit the manuscript for publication.

## Results

**Figure 1:**
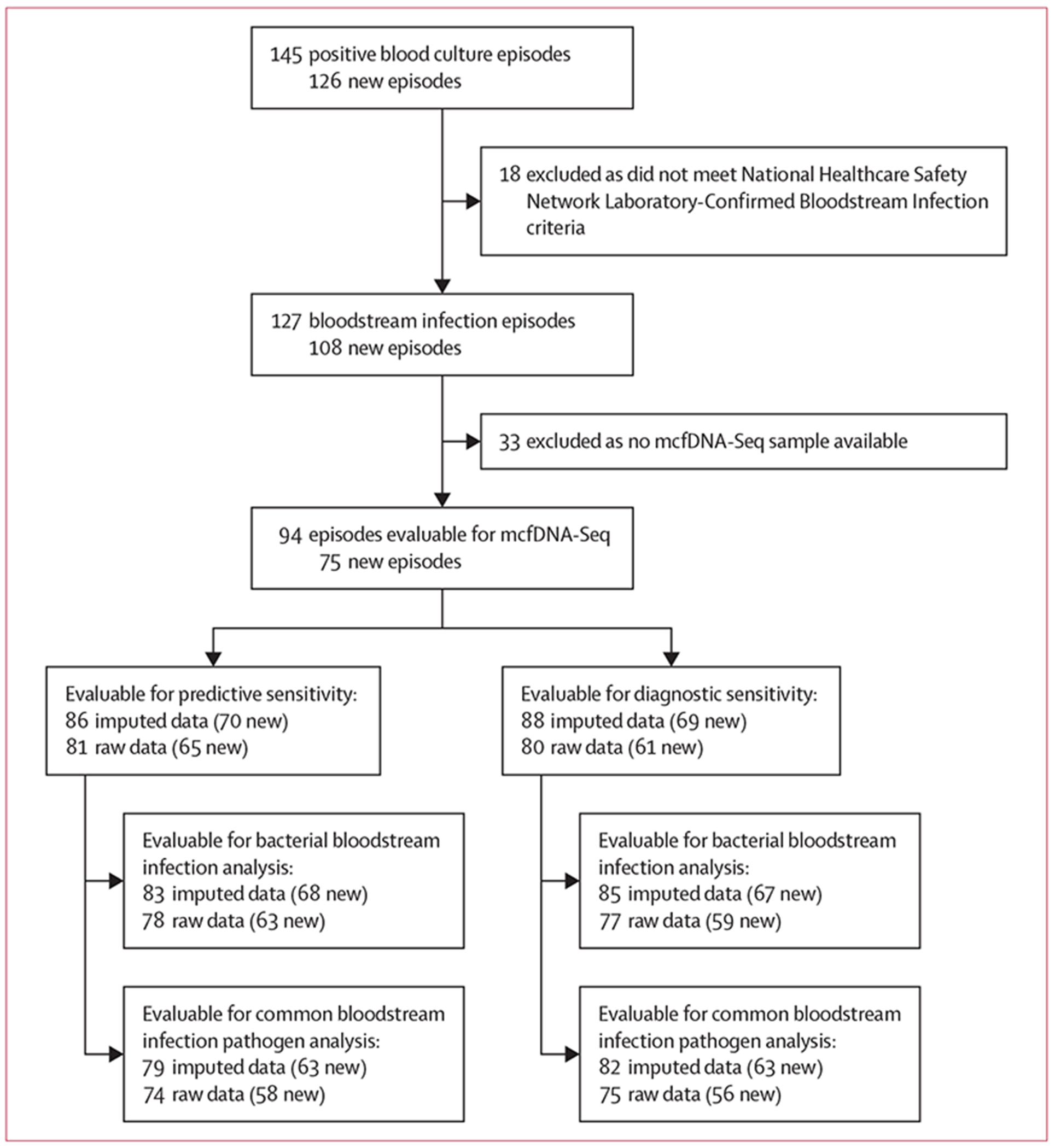
Flow diagram of bloodstream infection episodes and available samples Flowchart is per bloodstream infection episode; flowchart per participant is presented in the appendix (p 16). New episodes or samples refer to those from the completion phase. mcfDNA-Seq=microbial cell-free DNA metagenomic sequencing.

Between Aug 9, 2017, and Feb 28, 2022, 158 participants with leukaemia were enrolled in the study and provided samples on a median of 3·4 (IQR 2·6–4·3) days per week (appendix p 5); sampling frequency was not associated with clinical variables (appendix p 6). There were study disruptions between approximately Oct 20, 2018, and May 29, 2019, due to personnel limitations, and from March 15 to Aug 23, 2020, as a result of institutional COVID-19 pandemic research restrictions. Of 127 bloodstream infection episodes, 94 episodes in 60 participants had at least one evaluable mcfDNA-Seq plasma sample available (figure 1, table 1, appendix pp 7, 16).^18^ There were 162 evaluable control episodes (table 1).^18^

**Figure 2:**
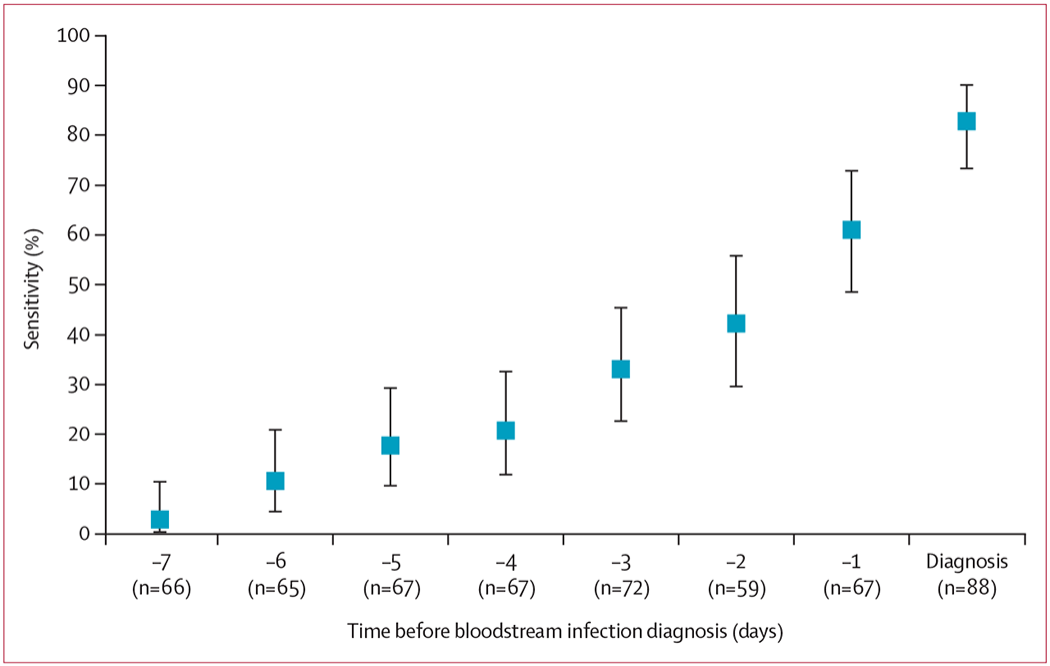
Sensitivity of microbial cell-free DNA metagenomic sequencing for predicting bloodstream infection by days before onset with logical imputation of missing data Figure includes consolidated data from pilot and completion phases. Bars denote 95% CIs. Diagnosis=sample collected on day 0 or day 1.

The pathogens identified by blood culture were also detected by mcfDNA-Seq during the a priori-defined predictive period (≤3 days before onset) in 30 (46%) of 65 evaluable new bloodstream infection episodes and at diagnosis in 49 (80%) of 61 evaluable new bloodstream infection episodes (appendix p 8). Consolidated predictive sensitivity (during the 3 days before bloodstream infection onset) comprising new evaluable episodes and those already reported^18^ was 51·9% (95% CI 40·5–63·1) for all bloodstream infection episodes, which is greater than favourable sensitivity, and 53·8% (42·2–65·2) for bacterial infection only. Consolidated diagnostic sensitivity was 81·3% (71·0–89·1%) for all bloodstream infection episodes and 83·1% (72·9–90·7) for bacterial infections only (table 2, appendix p 9). After applying a post-hoc, data-derived molecules-per-μL cutoff (≥140 molecules per μL) selected from receiver operating characteristic curve analysis (area under the receiver operating characteristic curve 0·73) of the consolidated data, predictive sensitivity remained at 51·9% (40·5–63·1) and diagnostic sensitivity was 80·7% (70·9–88·3%). Sensitivity was lowest at day −7 and increased daily until the day of diagnosis. Figure 2 shows the sensitivity by day for all organisms; the appendix (pp 9, 17–19) shows sensitivity data for all organisms and for bacteria only, using raw data or with logical imputation of missing values.

The generalised linear mixed model analysis showed that predictive sensitivity was highest in the 3 days before onset of infection, compared with days −7 to −4 (aOR 13·2 [95% CI 3·3–51·8]), and in participants with acute lymphoblastic leukaemia, compared with those with acute myeloid leukaemia (aOR 11·1 [1·7–74·2]; appendix p 10). Because detection of all clinically detected organisms in polymicrobial infections was required for success, sensitivity in such infections was lower than in monomicrobial infections (aOR 0·0 [0·0–0·2] for comparison to mono-microbial Gram-positive infections). By contrast, patient age, white blood cell or absolute neutrophil count, sepsis, time to positivity of blood cultures, and gastrointestinal acute GVHD did not significantly affect sensitivity. There were no missing data for any variables included in the generalised linear mixed model analysis.

Of 129 new control samples with no fever or infection within the 7 days before or after sample collection, 22 (17%) had at least one bacterium or yeast identified by mcfDNA- Seq, compared with six (18%) of 33 in the pilot phase. Consolidated specificity was 83% (95% CI 76–88) for any organism and 89% (83–93) for common bloodstream infection pathogens (table 3). Organisms identified in control samples were typically detected at concentrations much lower than those of expected bloodstream infection organisms in bloodstream infection samples (p<0·0001; appendix pp 11, 20–21), and most were not common bloodstream infection pathogens (appendix pp 12–13). Applying a post-hoc, data-derived molecules-per-μL cutoff (≥140 molecules per μL) to the consolidated data increased specificity for common bloodstream infection pathogens to

94% (89–97; table 3). Clinical false-positive results were positively associated with gastrointestinal disturbance (diarrhoea, abdominal pain, typhlitis, colitis, or gastrointestinal acute GVHD) alone (three [60%] of five *vs* 25 [16%] of 157; p=0·037) or combined with recent administration of high-dose cytarabine (eight [38%] of 21 *vs* 20 [14%] of 141; p=0·012), but not participant age, leukaemia type, white blood cell or neutrophil count, and HCT (appendix p 14).

Of the 80 consolidated bloodstream infection episodes with at least one evaluable sample available at diagnosis, 29 (36%) had an additional organism identified by plasma mcfDNA-Seq that was not identified by blood culture, including 22 (28%) with common bloodstream infection pathogens (appendix p 15). However, an untreated organism was detected in only eight (10%) episodes, including six (8%) with common bloodstream infection pathogens, mostly *Lactobacillus* spp associated with probiotic administration. Trajectory analysis suggested that these additional organisms were less likely than expected organisms to have an increasing DNA concentration during the days preceding bloodstream infection diagnosis (appendix p 22).

## Discussion

This study evaluated the sensitivity and specificity of mcfDNA-Seq for predicting impending bloodstream infections in immunocompromised children. The findings confirm and extend previous results by showing that the test is sensitive and specific for impending infection, and that the specificity might be further enhanced without affecting the sensitivity by using trajectory analysis and by excluding lower concentrations of bacterial DNA frag- ments.^18^ The consolidated predictive sensitivity during the 3 days before bloodstream infection onset (51·9%) was higher than the a priori protocol-defined target of 50% for potential use as a screening test. Valuable insights into interpreting mcfDNA-Seq in immunocompromised patients have been obtained by analysing negative-control samples. The specificity analyses suggest that pathogen DNA, especially at low concentrations or from organisms that do not commonly cause bloodstream infections in this population, does not always represent an existing or impending infection and that patients with gastrointestinal disease or recent high-dose cytarabine exposure are at highest risk for clinical false-positive results.

These findings are consistent with pilot data from this cohort,^18^ which showed predictive sensitivity of 75% in 16 bloodstream infection episodes, and from other small studies that evaluated the performance of mcfDNA-Seq for predicting infection in immunocompromised patients. Sensitivity estimates were slightly lower in the new dataset alone than in the composite dataset including the pilot data. A small observational study evaluating the prediction of bloodstream infections in adults in the intensive care unit by mcfDNA-Seq (16 episodes) estimated the predictive sensitivity as 87·5%.^16^ A larger study evaluating the prediction of invasive pulmonary fungal infections in adult patients undergoing HCT (39 episodes) estimated the predictive sensitivity as up to 37·5% in the week before diagnosis.^17^ Therefore, mcfDNA-Seq has the potential to predict fungal, bacterial, and DNA-viral infections in immunocompromised patients or those with critical illness.

The prospective design and prespecified analyses, masking of the testing laboratory, and large-scale parallel testing of control and bloodstream infection samples from the same population enabled robust evaluation of the predictive performance of mcfDNA-Seq. Parallel testing of bloodstream infection samples and control samples collected from the same participant group avoided the potential for incorporation bias that can affect clinical validation of microbiological tests in immunocompromised patients, as detected organisms can be falsely attributed to febrile neutropenia or to other non-specific syndromes.^27^ The estimation of sensitivity across multiple timepoints before bloodstream infection onset provided insight into optimal testing strategies and supports a previously proposed test frequency of 3 non-consecutive days per week.^14^

A favourable sensitivity of 50% for predicting bloodstream infections was selected a priori as being potentially acceptable to clinicians. This sensitivity was selected to mirror the bloodstream infection risk reduction due to antibacterial prophylaxis in this population and is similar to the sensitivity of serum galactomannan, which has been implemented for routine screening in high-risk pop- ulations.^10,12,28,29^ This suggests that implementing mcfDNA- Seq screening might be acceptable, depending on the cost and turnaround time. Adding other modalities, such as immune biomarkers, to mcfDNA-Seq might further increase sensitivity, but this has not been evaluated.

There are potential risks and benefits associated with a novel paradigm of pre-emptive treatment for impending bloodstream infections. The primary benefit would be the opportunity to abort bloodstream infection episodes before they become clinically apparent, which might prevent progression to severe sepsis, death, or other serious sequelae^1,2,4–7,30^ and reduce rates of hospitalisation, antibiotic exposure, and antibiotic resistance. These benefits are important because infection is the most important cause of non-relapse mortality in this population, and even survivors can be left with permanent neurocognitive or other organ dysfunction. However, the effectiveness of this paradigm has not been evaluated. Potential drawbacks include the initiation of antibiotic therapy for false-positive test results, the demands on institutional resources, and the financial costs of testing. Therefore, interventional studies that address these issues are needed before routine implementation can be recommended.

This study has some limitations. Although mcfDNA-Seq could identify the causative organism before an infection becomes clinically apparent, the imperfect sensitivity means that many would not be predicted, and pre-emptive antimicrobials might not prevent bloodstream infections in all predicted episodes. The presence of DNA from *Burkholderia* spp in many samples, and of non-bloodstream infection organisms in several others, suggests that caution is needed to avoid overtreatment and that initially restricting prediction and pre-emptive therapy to a subset of common bloodstream infection pathogens might be appropriate.^23^ Because this study did not evaluate mcfDNA-Seq prediction of antimicrobial resistance, treatment decisions might still rely on typical susceptibility patterns. Sensitivity during the diagnostic period might not represent true diagnostic sensitivity because the relevant samples could be collected before or after the time of clinical presentation. Blood samples were obtained opportunistically, so bias might have been introduced by sample availability. Plasma samples were collected through the central venous catheter, and peripheral blood might yield different results. Lastly, the sensitivity of mcfDNA-Seq in predicting other common infectious complications in immunocompromised patients—such as febrile neutropenia, pneumonia, or skin and soft tissue infections—has not been evaluated, so it is unknown whether these complications can also be predicted.

Together, these data suggest that a successful model of pre-emptive antibacterial therapy guided by regular screening with mcfDNA-Seq might prevent up to 50% of bloodstream infection episodes in children with cancer. A proposed clinical workflow implementation could comprise the following: discontinuing routine antibacterial prophylaxis; rapid turnaround mcfDNA-Seq testing, restricted to common bloodstream infection pathogens detected above a predefined threshold and performed on 3 non-consecutive days per week; initiating targeted antimicrobials and immediately collecting a repeat sample in response to a positive test; discontinuing antimicrobial therapy early if the repeat test before antimicrobial initiation is not supportive of impending infection; and administering a defined course of antimicrobial therapy for other episodes (potentially of shorter duration than that for bloodstream infections). To optimise specificity, a positive test might exclude common contaminants or very low concentrations of pathogen DNA, and the approach might best exclude patients with acute gastrointestinal GVHD or recent high-dose cytarabine exposure. This model would require an mcfDNA-Seq test with a very fast turnaround (ideally performed on site), requiring only a small blood sample and having a reasonable cost–benefit profile. Antimicrobial stewardship oversight would be paramount to ameliorate the risk of increased unnecessary antimicrobial use.

In conclusion, microbial cell-free DNA metagenomic sequencing can detect causative pathogens before the onset of bloodstream infection in profoundly immunocompromised patients. The a priori-defined favourable sensitivity of this approach warrants clinical trials of mcfDNA-Seq- guided pre-emptive therapy to prevent life-threatening infections in immunocompromised patients. Although the specificity is constrained by factors such as gastrointestinal barrier dysfunction and the broad range of potential pathogens, implementation strategies that account for our new understanding of these limitations could optimise the clinical use of this approach and lead to a paradigm shift in the care of this vulnerable population.

## Supplementary Material

1

## Figures and Tables

**Table 1:** Characteristics of bloodstream infection and control episodes

	Bloodstream infection (n=94)	Control episode (n=162)
Age, years	10·0 (6·2)	10·9 (6·1)

Leukaemia group		
Acute lymphoblastic leukaemia	28 (30%)	41 (25%)
Acute myeloid leukaemia	64 (68%)	117 (72%)
Mixed or other	2 (2%)	4 (2%)

Haematopoietic cell transplantation	39 (41%)	30 (19%)
Haploidentical	28 (30%)	17 (10%)
Matched sibling	4 (4%)	4 (2%)
Matched unrelated	7 (7%)	9 (6%)

Time since haematopoietic cell transplantation, days	179·8 (269·5)	149·6 (172·1)

Gastrointestinal acute graft-versus-host disease	10 (11%)	1 (1%)
Grade 1	1 (1%)	0
Grade 2	0	0
Grade 3	1 (1%)	0
Grade 4	8 (9%)	1 (1%)

White blood cell count	1·6 (4·3)	2·1 (3·1)

Absolute neutrophil count	765·6 (2354·4)	1077·0 (1722·5)
<100	74 (79%)	59 (36%)
≥100 to <500	4 (4%)	28 (17%)
≥500	16 (17%)	75 (46%)

Bloodstream infection organism group		
Gram-positive	37 (39%)	NA
Gram-negative	44 (47%)	NA
Yeast	3 (3%)	NA
Polymicrobial	10 (11%)	NA

Time to positivity, h	14·8 (7·5)	NA

Intensive care unit admission for sepsis	13 (14%)	NA

Data are n (%) or mean (SD). Table includes consolidated data from pilot and completion phases. Baseline characteristics are per bloodstream infection episode; baseline characteristics per participant are presented in the appendix (p 5). NA=not applicable.

**Table 2:** Sensitivity of microbial cell-free DNA metagenomic sequencing for predicting and detecting bloodstream infection

	Raw data			With logical imputation of missing values		
	Evaluable episodes	Positive	Sensitivity	Evaluable episodes	Positive	Sensitivity
All bloodstream infection episodes (n=94)						
Prediction (day −3 to −1)	81	42	51·9% (40·5–63·1)	86	44	51·2% (40·1–62·1)
Diagnosis (day 0 or 1)	80	65	81·3% (71·0–89·1)	88	73	83·0% (73·4–90·1)

Bacterial bloodstream infections (n=91)						
Prediction (day −3 to −1)	78	42	53·8% (42·2–65·2)	83	44	53·0% (41·7–64·1)
Diagnosis (day 0 or 1)	77	64	83·1% (72·9–90·7)	85	72	84·7% (75·3–91·6)

Common bloodstream infection pathogens (n=87)						
Prediction (day −3 to −1)	74	38	51·4% (39·4–63·1)	79	40	50·6% (39·1–62·1)
Diagnosis (day 0 or 1)	75	61	81·3% (70·7–89·4)	82	68	82·9% (73·0–90·3)

Data are n or % (95% CI). Table includes consolidated data from pilot and completion phases.

**Table 3:** Specificity of microbial cell-free DNA metagenomic sequencing in control samples

	Negative (n=162)	Specificity
Any organism*		
All positive tests	134	82·7% (76·0–88·2)
≥140 molecules per μL†	147	90·7% (85·2–94·7)

Common bloodstream infection pathogen‡		
All positive tests	144	88·9% (83·0–93·3)
≥140 molecules per μL†	152	93·8% (88·9–97·0)

Data are n or % (95% CI). Table includes consolidated data from pilot and completion phases.

* Excludes *Burkholderia* spp, moulds, viruses, and parasites.

† Organisms detected with a reported DNA concentration of 140 molecules per μL of plasma or higher.

‡ Includes all genera identified in more than 1% of central line-associated bloodstream infection episodes in children with cancer (appendix p 4).

## Data Availability

The study protocol is provided in the appendix (pp 24–52). Consent forms and other study-related documents are available by request to the corresponding author. Individual participant data are not available.

## Research in context

### Evidence before this study

We searched PubMed from database inception until May 4, 2025, using the terms (“cell free DNA” OR “cell-free DNA” OR “metagenomic sequencing”) AND infection AND (prediction OR predictive OR pre-emptive), to identify original studies evaluating microbial cell-free DNA metagenomic sequencing (mcfDNA-Seq) for prediction of bacterial or fungal infections. All titles were reviewed for relevance. We identified two small observational studies evaluating bloodstream infection prediction by mcfDNA-Seq (with 16 predictive episodes in each study), which estimated the predictive sensitivity as 87·5% during the week before bloodstream infection onset in adults in the intensive care unit and 75·0% during the 3 days before bloodstream infection onset in children with leukaemia (the pilot phase of this study). A larger study evaluated the prediction of invasive pulmonary fungal infections (39 episodes) in adult patients undergoing haematopoietic cell transplantation and estimated the predictive sensitivity as up to 37·5% in the week before diagnosis. Masking was reported in only one study. An updated search on Sept 8, 2025 yielded no new studies. No meta-analysis was performed.

### Added value of this study

This study confirms and extends the findings of previous studies by showing that the pathogens causing bloodstream infection can be identified by plasma mcfDNA-Seq before diagnosis and by identifying characteristics associated with improved sensitivity. The study also provides valuable insight into the specificity of mcfDNA-Seq in immunocompromised hosts by evaluating the performance of the test with a large group of samples collected from the same patient population in the absence of recent or incipient infection, and by assessing the clinical significance of additional organisms identified by mcfDNA-Seq at the time of bloodstream infection onset.

### Implications of all the available evidence

The combined available evidence shows that plasma mcfDNA-Seq can detect bloodstream infection pathogens in the days before onset of infection in immunocompromised children in around half of the episodes. Although the test also detects pathogen DNA from organisms of uncertain or no clinical significance, its clinical specificity could be improved by selecting a higher concentration threshold, attending to DNA concentration kinetics, and restricting analyses to common bloodstream infection organisms. Interventional studies to evaluate mcfDNA-Seq-guided pre-emptive therapy in immunocompromised hosts are warranted.
