# Supplementary material for: Predicting bloodstream infection by plasma cell-free metagenomic sequencing: a prospective cohort study
Source: Lancet Microbe. Author manuscript; Available in PMC 2026 May 8. (PMC13154368; doi:10.1016/j.lanmic.2025.101312)
Supplement: 1 [file NIHMS2164955-supplement-1.pdf]

THE LANCET
Microbe

Supplementary appendix

This appendix formed part of the original submission and has been peer reviewed.
We post it as supplied by the authors.

Supplement to: Wolf J, Goggin KP, Inaba Y, et al. Predicting bloodstream infection by plasma cell-free metagenomic sequencing: a prospective cohort study. *Lancet Microbe* 2026.
https://doi.org/10.1016/j.lanmic.2025.101312

Online Supplement

Table of Contents

Supplemental Methods 2

Supplemental Tables 4

eTable 1. Organisms classified as common BSI pathogens in children with cancer 4

eTable 2. Characteristics of study participants with and without bloodstream infection (*N* = 158)......... 5

eTable 3. Frequency of plasma sample collection according to clinical variables................................. 6

eTable 4. Episodes of positive blood cultures... 7

eTable 5. Sensitivity of mcfDNA-Seq for predicting and detecting bloodstream infection in new samples from the completion phase.. 8

eTable 6. Sensitivity of mcfDNA-Seq at each timepoint using raw and imputed data 9

eTable 7. Generalized linear mixed model analysis of characteristics associated with sensitivity for each BSI episode 10

eTable 8. Organisms detected by mcfDNA-Seq in control episode samples (*N* = 162) 11

eTable 9. Frequency of organisms causing BSI episodes vs. organisms detected by mcfDNA-Seq in control samples.. 12

eTable 10. Characteristics associated with pathogen detection in control episode samples (clinical false positives) 14

eTable 11. Characteristics of additional organisms detected by mcfDNA-Seq in diagnostic samples from BSI episodes 15

Supplemental Figures 16

eFigure 1. Participants included in analysis.. 16

eFigure 2. Sensitivity of mcfDNA-Seq for predicting bloodstream infection by days before onset (raw data).. 17

eFigure 3. Sensitivity of mcfDNA-Seq for predicting bacterial bloodstream infection by days before onset (with logical imputation of missing data) 18

eFigure 4. Sensitivity of mcfDNA-Seq for predicting bacterial bloodstream infection by days before onset (raw data) 19

eFigure 5. Violin plot of concentrations of expected microbial DNA detected by mcfDNA-Seq in BSI-related samples compared to concentrations in control samples 20

eFigure 6. Receiver operating characteristic curve for MPM values during the predictive period (Day –3 to –1) in BSI samples vs. control samples.. 21

eFigure 7. Trajectory of mcfDNA-Seq–detected DNA concentrations for expected vs. additional organisms in BSI-related samples 22

Supplemental References..23

Study Protocol 24

Supplemental Methods

Study Eligibility Criteria

Inclusion Criteria:

1. Under 25 years of age at time of study enrollment
2. Undergoing care for cancer at St. Jude
3. In a category of patients who are considered by the investigator to be at high risk of infection
4. Expected to receive care at St. Jude for at least 7 days

Exclusion Criteria:

1. Any condition that would, in the opinion of the investigator, place the subject at an unacceptable risk of injury or render the subject unable to meet the requirements of the protocol

Clinical Care Standards

The frequency of collection of blood for hematologic testing and, consequently, secondary retention for research testing in this study was determined by the treating clinicians. According to institutional practice, blood for hematology testing is typically drawn from an indwelling central venous catheter (CVC).

Patients presenting with fever, sepsis, or suspected infection had blood collected for culture from all lumens of indwelling CVCs. Although peripheral blood culture was also recommended for all new episodes of fever or suspected infection, it was performed at the discretion of the treating clinician. The choice of empiric antimicrobial therapy was determined by clinical risk stratification and by the patient's history of infection or colonization with antimicrobial-resistant organisms.[1] Empiric antimicrobial therapy was continued, modified, or discontinued according to the clinical response, the diagnosis of clinical or microbiological infection, and the severity of the initial presentation. Clinical care standards for preventing and managing infections did not change significantly during the study period.

Microbial Cell-Free DNA Sequencing Method

Sample handling and preparation

Upon receipt at the testing laboratory (Karius Inc., Redwood City, CA), controls for carry-over, sequencing bias, metagenomic sequencing quality, and sample mix-ups were added to the sample. In addition, two types of batch controls were run alongside patient samples. Four replicates of environmental control samples containing buffer instead of plasma were processed in parallel with patient samples from accessioning to report generation. Environmental controls were used to monitor microbial DNA signals arising from the background at the time of batch processing. The estimated taxon abundances from the environmental control samples within the batch were combined to parameterize a model of read abundance arising from the environment, with variations driven by counting noise. Two assay controls, each containing four distinct species ranging in GC content from 30% to 65% at high or low concentrations were included in each batch. All four spiked microorganisms and no others had to be detected within a specified MPM range in both assay controls for the sample to pass the final quality control inspection. Proprietary chemistries were used to enrich samples for mcfDNA without preselecting pathogens to test.

Sequencing

Automated DNA extraction and sequencing library preparation protocols were optimized for high speed and low pathogen bias. Single-end, 56-cycle sequencing was performed on NextSeq 500 instruments (Illumina, San Diego, CA) with an average of >20 million reads/sample. Double-unique dual indexes were used to ensure robust sample de-multiplexing. Synthetic DNA molecules that contain 16 degenerate bases were spiked into plasma at a specific molar concentration to monitor the yield and quality of the entire workflow. By counting the number of these distinctive molecules in the sequencing data obtained from each sample, the whole assay yield was monitored and the concentration of each microbial cfDNA species in the original plasma aliquot determined. Samples for which a minimum number of these molecule reads failed to be achieved for any reason (for example, poor yield in any workflow step, poor sequencing quality, or failure to properly de-multiplex a sample) resulted in a one-time requeue.

Analysis pipeline

Sequencing data were processed using a proprietary analytical pipeline, and microbial reads were aligned to a database comprising >20,000 curated assemblies from >16,000 species, of which >1500 taxa, including bacteria, DNA viruses, fungi, and parasites, are in the clinical reportable range (CRR) of the test. Organisms in the CRR were selected as follows: A candidate list of human pathogens was generated by two board-certified infectious disease physicians by including (i) DNA viruses, bacteria, mycobacteria, fungi, and parasites from the standard textbooks and other relevant references, (ii) organisms in the pathogen database referenced in published case reports, and (iii) reference genomes sequenced from human clinical isolates, with publications supporting their potential pathogenicity. Organisms from the above list that were associated with high-quality reference genomes, as determined by our reference database quality control process, were used to narrow the range further. Finally, organisms at risk for generating common false-positive calls because of sporadic environmental contamination were removed from the CRR. The current CRR is available at https://kariusdx.com/the-karius-test/pathogen-list/.

Statistical Considerations

The study used a Simon's optimal two-stage design with minimal acceptable predictive sensitivity of 30% and favorable predictive sensitivity of 50%. Favorable sensitivity is the *a priori*–defined minimum sensitivity that might justify further investigation. It was selected to mirror the approximate efficacy of antibacterial prophylaxis in children with leukemia on the assumption that a test that was able to predict and enable pre-emptive therapy for a similar proportion of BSI episodes could be acceptable to clinicians.[2] As specified in the study protocol, the overall target sample size was 100 BSI events, with the aim of estimating the sensitivity and specificity of the test to a 95% confidence interval of ±10%, assuming a sensitivity of ~50% and specificity of ~80%.

For estimating sensitivity, logical imputation was used to impute missing data where possible, as described previously.[3] Positive mcfDNA-Seq results were carried forward to subsequent days that had missing values, and negative mcfDNA-Seq results were carried backward to prior days that had missing values. Results of analyses performed using both raw data and data with logical imputation applied are provided.

For evaluation of the association between episode or participant characteristics and predictive sensitivity, a binomial distribution with a logit link function was used as the base for the generalized linear mixed model (GLMM), with test positivity (i.e., whether the mcfDNA-Seq detected the BSI pathogen[s]) serving as the binary outcome in the model. Clinical covariates of interest were included as fixed effects to estimate their associations with mcfDNA-Seq test positivity. A random intercept for each BSI episode was included to account for correlation due to repeated measures within the same episode, and clinical covariates were modeled as predictors. Clinical covariates evaluated were: sample collection timepoint, age, leukemia group, white blood cell count, absolute neutrophil count, hematopoietic cell transplantation, sepsis requiring ICU admission, gastrointestinal acute graft versus host disease, organism group, and time to positivity of diagnostic blood cultures.

Specificity was defined as the proportion of evaluable control samples in which any bacteria or yeasts were detected by mcfDNA-Seq. The primary specificity analysis covered bacteria or yeasts; parasites, molds, and viruses were excluded *a priori*. After a review of mcfDNA-Seq data showed that *Burkholderia cepacia* was identified in 35 of 162 control samples (21.6%) and there was no increase in detection of this organism at the laboratory performing the mcfDNA-Seq, suggesting possible point sample contamination in the local hospital laboratory, *B. cepacia* was excluded from analyses of specificity.

Supplemental Tables

eTable 1. Organisms classified as common BSI pathogens in children with cancer

Organism
**Gram-positive bacteria**
Bacillus spp.
Clostridium spp.
Corynebacterium jeikeium
Enterococcus spp.
Lactobacillus spp.
Rothia spp.
Staphylococcus spp.
Streptococcus spp.
**Gram-negative bacteria**
Citrobacter spp.
Escherichia coli
Klebsiella spp.
Pseudomonas spp.
Stenotrophomonas maltophilia
**Fungi**
Candida spp.

Organisms identified in more than 1% of episodes of central line–associated bloodstream infection in children with cancer, as recorded by the Children's Hospital Association Childhood Cancer & Blood Disorders Network, August 2013–December 2015.[4]

eTable 2. Characteristics of study participants with and without bloodstream infection ($N = 158$)

	BSI (N = 60)		No BSI (N = 98)		All participants (N = 158)	
**Characteristic**	**n**	**%**	**n**	**%**	**n**	**%**
**Age in years, median (IQR)**	9.7	(5.0–15.0)	11.2	(6.4–14.9)	10.4	(5.6–14.9)
**Sex**						
Female	31	(51.7%)	42	(42.9%)	73	(46.2%)
Male	29	(48.3%)	56	(57.1%)	85	(53.8%)
**Race (self-reported)**						
White	43	(71.7%)	66	(67.3%)	109	(69.0%)
Black	8	(13.3%)	17	(17.3%)	25	(15.8%)
Asian	4	(6.7%)	3	(3.1%)	7	(4.4%)
American Indian/Alaskan Native	3	(5.0%)	3	(3.1%)	6	(3.8%)
Other or multiple specified	2	(3.3%)	9	(9.2%)	11	(7.0%)
**Ethnicity**						
Hispanic	20	(33.3%)	30	(30.6%)	50	(31.6%)
Non-Hispanic	40	(66.7%)	68	(69.4%)	108	(68.4%)
**Leukemia type**						
ALL	19	(31.7%)	35	(35.7%)	54	(34.2%)
AML	40	(66.7%)	61	(62.2%)	101	(63.9%)
Mixed/other	1	(1.7%)	2	(2%)	3	(1.9%)
**Hematopoietic cell transplantation**	33	(55.0%)	50	(51.0%)	83	(52.5%)

BSI, evaluable bloodstream infection during study period; ALL, acute lymphoblastic leukemia; AML, acute myeloid leukemia; IQR, interquartile range. Table includes consolidated data from pilot and completion phases.

eTable 3. Frequency of plasma sample collection according to clinical variables

	Sample frequency (days/week)		
**Variable**	**Median**	**IQR**	***P***
**Age group**			0.63
≤10 years	3.36	(2.7–4.3)	
>10 years	3.38	(2.4–4.3)	
**Sex**			0.36
Female	3.36	(2.6–4.2)	
Male	3.38	(2.7–4.3)	
**Leukemia group**			0.87
ALL	3.31	(1.7–4.8)	
AML	3.38	(2.8–4.1)	
**HCT**			0.43
Yes	3.43	(2.6–4.2)	
No	3.36	(2.7–4.3)	

IQR, interquartile range; HCT, hematopoietic cell transplantation

eTable 4. Episodes of positive blood cultures

**Episode outcome**	**n (*N* = 145)**	**%**
Included	94	64.8
NHSN-defined contaminant	18	12.4
No evaluable sample available		
During study pause	17	11.7
Sample processing delayed > 7 days	1	0.7
No sample available	8	5.5
Other	7	4.8

NHSN, National Healthcare Safety Network[5]

eTable 5. Sensitivity of mcfDNA-Seq for predicting and detecting bloodstream infection in new samples from the completion phase

	Raw data				With logical imputation of missing values			
	Evaluable episodes	Positive	Sensitivity	95% CI	Evaluable episodes	Positive	Sensitivity	95% CI
**All BSI episodes (n = 75)**								
Prediction (day −3 to −1)	65	30	46.2%	(33.7-59.0)	70	32	45.7%	(33.7-58.1)
Diagnosis (day 0 or 1)	61	49	80.3%	(68.2-89.4)	69	57	82.6%	(71.6-90.7)
**Bacterial BSI (n = 73)**								
Prediction (day −3 to −1)	63	30	47.6%	(34.9-60.6)	68	32	47.1%	(34.8-59.6)
Diagnosis (day 0 or 1)	59	48	81.4%	(69.1-90.3)	67	56	83.6%	(72.5-91.5)
**Common BSI pathogens (n = 68)**								
Prediction (day −3 to −1)	58	26	44.8%	(31.7-58.5)	63	28	44.4%	(31.9-57.5)
Diagnosis (day 0 or 1)	56	45	80.4%	(67.6-89.8)	63	52	82.5%	(70.9-90.9)

BSI, bloodstream infection; 95% CI, 95% confidence interval

eTable 6. Sensitivity of mcfDNA-Seq at each timepoint using raw and imputed data

	Raw data				With logical imputation			
Sample day	Evaluable episodes	Positive tests	% positive	95% CI	Evaluable episodes	Positive tests	% positive	95% CI
**All BSI episodes**								
Day −7	28	2	7.1	(0.9–23.5)	66	2	3.0	(0.4−10.5)
Day −6	14	5	35.7	(12.8−64.9)	65	7	10.8	(4.4−20.9)
Day −5	22	9	40.9	(20.7−63.6)	67	12	17.9	(9.6−29.2)
Day −4	20	7	35.0	(15.4−59.2)	67	14	20.9	(11.9−32.6)
Day −3	44	16	36.4	(22.4−52.2)	72	24	33.3	(22.7−45.4)
Day −2	28	14	50.0	(30.6−69.4)	59	25	42.4	(29.6−55.9)
Day −1	50	30	60.0	(45.2−73.6)	67	41	61.2	(48.5−72.9)
Day 0	72	59	81.9	(71.1−90.0)	83	68	81.9	(72.0−89.5)
Day 1	25	21	84.0	(63.9−95.5)	79	75	94.9	(87.5−98.6)
Diagnosis (day 0 or 1)	80	65	81.2	(71.0−89.1)	88	73	83.0	(73.4−90.1)
Predictive (day −3 to −1)	80	41	51.2	(39.8−62.6)	86	44	51.2	(40.1−62.1)
**All bacterial BSI episodes**								
Day −7	28	2	7.1	(0.9−23.5)	63	2	3.2	(0.4−11.0)
Day −6	14	5	35.7	(12.8−64.9)	62	7	11.3	(4.7−21.9)
Day −5	21	9	42.9	(21.8−66.0)	64	12	18.8	(10.1−30.5)
Day −4	19	7	36.8	(16.3−61.6)	64	14	21.9	(12.5−34.0)
Day −3	43	16	37.2	(23.0−53.3)	69	24	34.8	(23.7−47.2)
Day −2	26	13	50.0	(29.9−70.1)	55	24	43.6	(30.3−57.7)
Day −1	48	31	64.6	(49.5−77.8)	64	41	64.1	(51.1−75.7)
Day 0	69	58	84.1	(73.3−91.8)	80	67	83.8	(73.8−91.1)
Day 1	24	21	87.5	(67.6−97.3)	77	74	96.1	(89.0−99.2)
Diagnosis (day 0 or 1)	77	64	83.1	(72.9−90.7)	85	72	84.7	(75.3−91.6)
Predictive (day −3 to −1)	77	41	53.2	(41.5−64.7)	83	44	53.0	(41.7−64.1)

Table includes consolidated data from pilot and completion phases.

eTable 7. Generalized linear mixed model analysis of characteristics associated with sensitivity for each BSI episode

Variable	Unadjusted odds ratio			Adjusted odds ratio		
	OR	95% CI	*P*	aOR	95% CI	*P*
**Sample collection timepoint**						
Day −7 to day −4	Reference			Reference		
Day −3 to day −1	14.6	(3.7–58.7)	**0.00015**	13.2	(3.3–51.8)	**0.00023**
Day 0 to day 1	1033.8	(70.8–15097.5)	**<0.0001**	1059.58	(68.09–16488.7)	**<0.0001**
**Age, years**	0.9	(0.9−1.0)	0.066	-		-
**Leukemia group**						
AML	Reference			Reference		
ALL	3.5	(1.6–7.8)	**0.0025**	11.1	(1.7–74.)	**0.013**
**White blood cell count (cells/mm^3)**	1.0	(0.9−1.1)	0.57	-		-
**Absolute neutrophil count**						
≥ 500 cells/mm^3	Reference			Reference		
< 500 cells/mm^3	0.7	(0.3−2.0)	0.49	-		-
**Hematopoietic cell transplantation**						
Did not receive	Reference			Reference		
Received	2.2	(1.0−4.8)	**0.047**	3.5	(0.6−19.7)	0.15
**Sepsis**						
Absent	Reference			Reference		
Present	0.5	(0.2−1.4)	0.18	-		-
**Gastrointestinal aGVHD**						
Absent	Reference			Reference		
Present	2.7	(0.8−8.8)	0.11	-		-
**Organism group**						
Gram positive bacterial	Reference			Reference		
Gram-negative bacterial	0.5	(0.2−1.0)	0.064	0.8	(0.2−4.1)	0.77
Fungal	0.3	(0.03−2.0)	0.20	0.03	(0.0−8.4)	0.22
Polymicrobial	0.06	(0.01−0.5)	**0.0060**	0.0	(0.0−0.2)	**0.0070**
**Minimum time to positivity, minutes**	1.0	(1.0−1.1)	0.16	-		-

OR, odds ratio; CI, confidence interval; aOR, adjusted odds ratio; aGVHD, acute graft-versus-host disease at time of sampling; sepsis, evidence of sepsis or septic shock requiring ICU admission within 24 hours of BSI onset. Variables with univariate $P < 0.05$ were included in the multivariate analysis. Table includes consolidated data from pilot and completion phases.

eTable 8. Organisms detected by mcfDNA-Seq in control episode samples ($N = 162$)

Episode	Organism	MPM
PQ101:1	***Klebsiella pneumoniae***	112
	Clostridium perfringens	99
PQ104:1	*Veillonella parvula*	41
PQ118:1	*Finegoldia magna*	129
PQ130:1	*Helicobacter pylori*	38
	Lactobacillus fermentum	25
PQ132:1	*Neisseria sicca*	609
PQ139:1	***Escherichia coli***	85
PQ148:1	*Atopobium vaginae*	106
PQ174:1	*Aspergillus fumigatus*	47
PQ183:1	***Klebsiella variicola***	139
PQ183:2	***Staphylococcus epidermidis***	1118
	Enterobacter cloacae complex	388
PQ190:1	***Streptococcus oralis***	631
	Pseudomonas aeruginosa	288
PQ192:2	*Leuconostoc mesenteroides*	106
PQ197:1	***Streptococcus mitis***	329
	Lactobacillus fermentum	157
PQ203:1	***Enterococcus faecium***	96
PQ205:1	*Acinetobacter baumannii*	27
PQ205:2	***Pseudomonas citronellolis***	21
PQ206:1	***Stenotrophomonas maltophilia***	3765
	Escherichia coli	3680
	Staphylococcus equorum	151
PQ211:1	***Escherichia coli***	539
PQ223:1	***Pseudomonas pseudoalcaligenes***	124
PQ225:2	*Bacteroides fragilis*	741
PQ226:1	***Escherichia vulneris***	270
	Leclercia adecarboxylata	142
	Erwinia gerundensis	124
	Enterobacter cloacae complex	115
	Pantoea agglomerans	73
	Aureobasidium pullulans	55
PQ229:2	*Erwinia billingiae*	478
	Pantoea agglomerans	208
PQ230:2	*Achromobacter ruhlandii*	104
	Acetobacter nitrogenifigens	27
PQ231:2	***Staphylococcus epidermidis***	2315
	Bacteroides fragilis	140
PQ236:1	*Aspergillus glaucus*	37
PQ238:2	***Escherichia coli***	363
PQ239:1	*Prevotella melaninogenica*	153
	Streptococcus infantis	61
PQ239:2	*Prevotella melaninogenica*	178
	Capnocytophaga sputigena	97
PQ241:1	***Escherichia coli***	758
PQ241:2	***Escherichia coli***	192

Bolded organism names are classified as common BSI pathogens. Table includes consolidated data from pilot and completion phases.

eTable 9. Frequency of organisms causing BSI episodes vs. organisms detected by mcfDNA-Seq in control samples

	BSI episodes (*N* = 94)		Control samples (*N* = 162)	
**Organism**	**n**	**% of episodes**	**n**	**% of samples**
Escherichia coli	21	(22.3%)	6	(3.7%)
**Coagulase-negative *Staphylococcus spp.***	17	(18.1%)	3	(1.9%)
Coagulase-negative *Staphylococcus sp.*	3	(3.2%)	0	(0.0%)
Staphylococcus epidermidis	13	(13.8%)	2	(1.2%)
Staphylococcus equorum	0	(0.0%)	1	(0.6%)
Staphylococcus haemolyticus	1	(1.1%)	0	(0.0%)
Klebsiella pneumoniae/variicola	13	(13.8%)	2	(1.2%)
**Viridans group *Streptococcus spp.***	9	(9.6%)	3	(1.9%)
Streptococcus infantis	0	(0.0%)	1	(0.6%)
Streptococcus mitis	0	(0.0%)	1	(0.6%)
Streptococcus oralis	0	(0.0%)	1	(0.6%)
Streptococcus sanguis	1	(1.1%)	0	(0.0%)
Viridans group *Streptococcus sp.*	8	(8.5%)	0	(0.0%)
Rothia mucilaginosa	6	(6.4%)	0	(0.0%)
Enterococcus faecium	6	(6.4%)	1	(0.6%)
Lactobacillus spp.	4	(4.3%)	2	(1.2%)
Lacticaseibacillus rhamnosus	1	(1.1%)	0	(0.0%)
Lactobacillus fermentum	0	(0.0%)	2	(1.2%)
Lactobacillus rhamnosus	1	(1.1%)	0	(0.0%)
Lactobacillus sp.	2	(2.1%)	0	(0.0%)
Pseudomonas aeruginosa	4	(4.3%)	1	(0.6%)
***Enterobacter cloacae* complex**	4	(4.3%)	2	(1.2%)
Candida spp.	3	(3.2%)	0	(0.0%)
Candida glabrata	1	(1.1%)	0	(0.0%)
Candida guillermondii	1	(1.1%)	0	(0.0%)
Candida krusei	1	(1.1%)	0	(0.0%)
Acinetobacter baumannii	2	(2.1%)	1	(0.6%)
Enterococcus gallinarum	2	(2.1%)	0	(0.0%)
Enterococcus faecalis	2	(2.1%)	0	(0.0%)
Fusobacterium nucleatum	2	(2.1%)	0	(0.0%)
Capnocytophaga spp.	1	(1.1%)	1	(0.6%)
Capnocytophaga sp.	1	(1.1%)	0	(0.0%)
Capnocytophaga sputigena	0	(0.0%)	1	(0.6%)
Bacillus cereus	1	(1.1%)	0	(0.0%)
Clostridium ramnosum	1	(1.1%)	0	(0.0%)
Stenotrophomonas maltophilia	1	(1.1%)	1	(0.6%)
Raoultella ornithinolytica	1	(1.1%)	0	(0.0%)
Corynebacterium jeikeium	1	(1.1%)	0	(0.0%)
Staphylococcus aureus	1	(1.1%)	0	(0.0%)
Leuconostoc sp.	1	(1.1%)	0	(0.0%)
Neisseria flavescens	1	(1.1%)	0	(0.0%)
Trichosporon asahii	1	(1.1%)	0	(0.0%)
Aureobasidium pullulans	0	(0.0%)	1	(0.6%)
Pseudomonas spp. (non-*aeruginosa*)	0	(0.0%)	2	(1.2%)
Pseudomonas citronellolis	0	(0.0%)	1	(0.6%)
Pseudomonas pseudoalcaligenes	0	(0.0%)	1	(0.6%)
Prevotella melaninogenica	0	(0.0%)	2	(1.2%)
Clostridium perfringens	0	(0.0%)	1	(0.6%)
Achromobacter ruhlandii	0	(0.0%)	1	(0.6%)
Aspergillus spp.	0	(0.0%)	2	(1.2%)
Pantoea agglomerans	0	(0.0%)	2	(1.2%)
Leclercia adecarboxylata	0	(0.0%)	1	(0.6%)
Erwinia gerundensis	0	(0.0%)	1	(0.6%)
Leuconostoc mesenteroides	0	(0.0%)	1	(0.6%)
Atopobium vaginae	0	(0.0%)	1	(0.6%)
Bacteroides fragilis	0	(0.0%)	2	(1.2%)
Finegoldia magna	0	(0.0%)	1	(0.6%)
Erwinia billingiae	0	(0.0%)	1	(0.6%)
Helicobacter pylori	0	(0.0%)	1	(0.6%)
Neisseria sicca	0	(0.0%)	1	(0.6%)
Veillonella parvula	0	(0.0%)	1	(0.6%)

	**BSI episodes (*N* = 94)**		**Control samples (*N* = 162)**	
**Organism**	**n**	**% of episodes**	**n**	**% of samples**
Acetobacter nitrogenifigens	0	(0.0%)	1	(0.6%)
Escherichia vulneris	0	(0.0%)	1	(0.6%)

Bolded organism names are classified as common BSI pathogens in children with cancer. Table includes consolidated data from pilot and completion phases.

eTable 10. Characteristics associated with pathogen detection in control episode samples (clinical false positives)

Characteristic	Bacteria or yeast DNA detected				
	No (N = 134)		Yes (N = 28)		*P*
**Age, median (IQR)**	10.9	(5.6−15.0)	9.9	(6.0−14.3)	0.49
**Leukemia group, n (%)**					0.38
ALL	32	(23.9%)	9	(32.1%)	
AML	99	(73.9%)	18	(64.3%)	
Mixed/other	3	(2.2%)	1	(3.6%)	
**WBC, median (IQR)**	1.1	(0.3−2.3)	1.4	(0.5−2.2)	0.72
**ANC, median (IQR)**	398.5	(10.0−1247.5)	350	(27.5−1707.5)	0.49
**Allogeneic HCT, n (%)**	25	(18.6%)	5	(17.1%)	0.20
None	109	(81.3%)	23	(82.1%)	
Haploidentical	14	(10.4%)	3	(10.7%)	
Matched sibling	2	(1.5%)	2	(7.1%)	
Matched unrelated	9	(6.7%)	0	(0.0%)	
**Days since HCT, median (IQR)**	69	(27.0−214.0)	111	(93.0−124.0)	0.64
**HD ARA-C or GI disturbance, n (%)**	13	(9.7%)	8	(28.6%)	**0.012**
Any GI disturbance, n (%)	2	(1.5%)	3	(10.7%)	**0.037**
HD ARA-C, n (%)	11	(8.2%)	6	(21.4%)	0.081

ALL, acute lymphoblastic leukemia; AML, acute myeloid leukemia; IQR, interquartile range; WBC, white blood cell count; ANC, absolute neutrophil count; HCT, hematopoietic cell transplantation; GI disturbance, any of diarrhea, abdominal pain, typhlitis, colitis, or acute gastrointestinal graft vs. host disease; HD ARA-C, high-dose cytarabine. Table includes consolidated data from pilot and completion phases.

eTable 11. Characteristics of additional organisms detected by mcfDNA-Seq in diagnostic samples from BSI episodes

Episode	Expected organism(s)	Additional organism(s)	MPM	Effective antimicrobial
PQ109:1	*Enterococcus faecium*	*Staphylococcus epidermidis*	763,462	Vancomycin
PQ109:2	*Enterococcus faecium*	*Staphylococcus epidermidis*	15,517	Vancomycin
PQ109:4	Coagulase-negative *Staphylococcus spp.*	*Lactobacillus acidophilus*	10,883	Penicillin
		Lactobacillus casei	9970	Penicillin
		Streptococcus thermophilus	62,860	Vancomycin
PQ109:5	*Pseudomonas aeruginosa*	***Lactobacillus acidophilus***	**15,073**	**None**
		Lactobacillus casei	**4305**	**None**
		Streptococcus thermophilus	14,600	Meropenem/cefepime
PQ110:1	*Rothia mucilaginosa*	*Streptococcus mitis*	16,090	Vancomycin
		Mucor velutinosus*	**1745**	**None**
PQ120:1	*Corynebacterium jeikeium*	*Staphylococcus epidermidis*	182	Vancomycin
		Aspergillus oryzae	1278	Posaconazole
PQ120:2	*Escherichia coli*	***Staphylococcus epidermidis***	**496**	**None**
PQ121:1	*Escherichia coli; Rothia mucilaginosa*	*Streptococcus mitis*	698	Vancomycin
PQ123:1	*Enterococcus faecium*	*Fusobacterium nucleatum*	5470	Linezolid
		Haemophilus parainfluenzae	2476	Cefepime
		Aspergillus flavus	1615	Voriconazole/micafungin
		Aspergillus oryzae	505	Voriconazole/micafungin
PQ124:1	*Candida glabrata*	*Staphylococcus epidermidis*	16,034	Vancomycin
PQ124:2	*Staphylococcus epidermidis*	*Candida glabrata*	616	Micafungin
		Enterococcus faecium	79,845	Vancomycin
PQ128:1	*Staphylococcus haemolyticus; Candida guillermondii*	*Streptococcus thermophilus*	715	Vancomycin
PQ129:1	*Enterococcus gallinarum*	*Candida albicans*	5004	Micafungin
		Candida glabrata	326	Micafungin
PQ138:1	*Trichosporon asahii*	***Lactobacillus rhamnosus***	**683**	**None**
PQ161:1	Coagulase-negative *Staphylococcus spp.*	***Klebsiella variicola***	**1854**	**None**
		Trichoderma atroviride	144	Micafungin
PQ164:1	*Enterobacter cloacae*	*Lactobacillus acidophilus*	552	Meropenem
		Lactobacillus casei	550	Meropenem
		Lactobacillus plantarum	857	Meropenem
		Rothia mucilaginosa	2991	Meropenem
		Streptococcus mitis	11,248	Meropenem
		Abiotrophia defectiva	634	Meropenem
		Granulicatella adiacens	863	Meropenem
PQ171:1	*Rothia mucilaginosa*	***Enterococcus gallinarum***	**211**	**None**
PQ175:1	*Staphylococcus epidermidis*	*Bifidobacterium animalis*	2892	Vancomycin
		Streptococcus thermophilus	2189	Vancomycin
		Lactobacillus plantarum	**1951**	**None**
		Lactobacillus acidophilus	**5938**	**None**
PQ176:1	*Lactobacillus sp.*	*Bifidobacterium animalis*	2620	Vancomycin/penicillin
PQ186:1	*Capnocytophaga sp.*	***Bacteroides fragilis***	**744**	**None**
		Fusobacterium nucleatum	2813	Cefepime
PQ203:1	*Enterococcus faecium*	*Trichosporon asahii*	1824	Posaconazole
PQ206:1	*Clostridium ramosum; Staphylococcus epidermidis*	*Streptococcus oralis*	2178	Cefepime
PQ207:1	Viridans group *Streptococcus sp.*	*Enterobacter cloacae*	661	Cefepime
PQ216:1	Viridans group *Streptococcus sp.*	*Gemella haemolysans*	2129	Vancomycin
PQ226:1	*Klebsiella pneumoniae*	*Escherichia coli*	50,057	Cefepime
PQ229:2	*Klebsiella pneumoniae*	*Streptococcus mitis*	224	Cefepime/ceftriaxone
PQ242:1	Coagulase-negative *Staphylococcus spp.*	*Escherichia coli*	696	Cefepime/levofloxacin
PQ244:1	*Neisseria flavescens*	*Neisseria mucosa*	2228	Cefepime
PQ246:1	*Enterococcus faecalis; Lactobacillus rhamnosus*	*Candida tropicalis*	206	Micafungin

Effective antimicrobial was defined as having received ≥3 days of an antimicrobial expected to be active against the organism based on local epidemiology or published data. Bolded names are organisms that were not treated by empiric therapy; *Participant with *Mucor spp.* cfDNA detected did not develop subsequent invasive fungal infection. Table includes consolidated data from pilot and completion phases.

Supplemental Figures

eFigure 1. Participants included in analysis

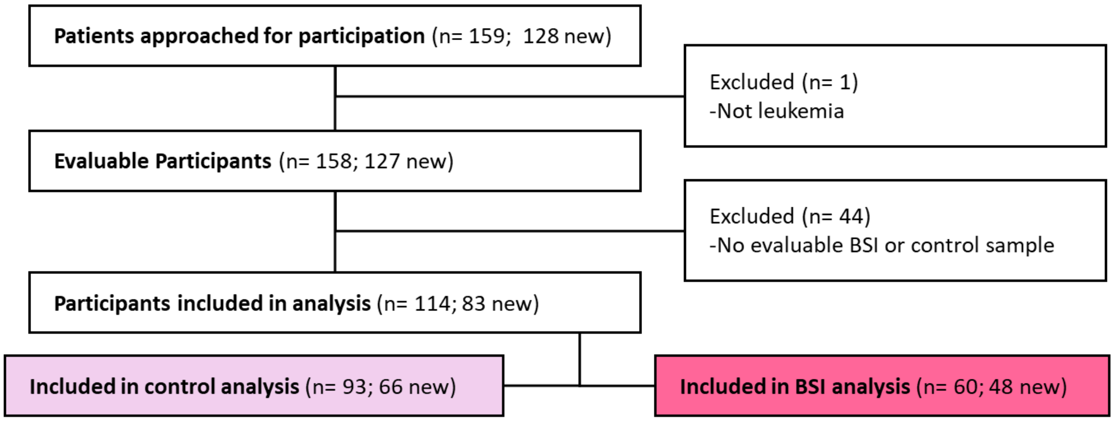

.

eFigure 2. Sensitivity of mcfDNA-Seq for predicting bloodstream infection by days before onset (raw data)

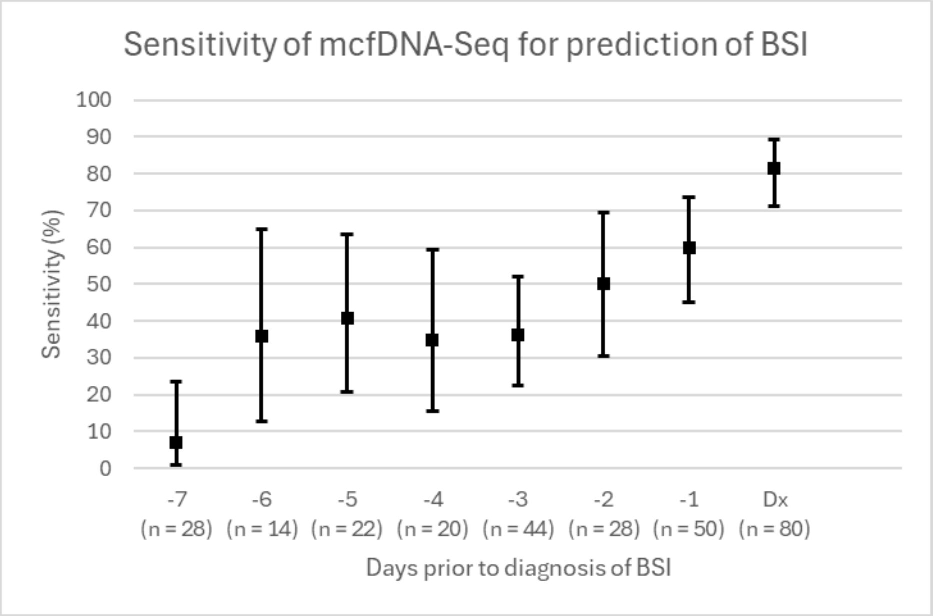

Dx, sample collected on day 0 or day +1; BSI, bloodstream infection. Figure includes consolidated data from pilot and completion phases.

eFigure 3. Sensitivity of mcfDNA-Seq for predicting bacterial bloodstream infection by days before onset (with logical imputation of missing data)

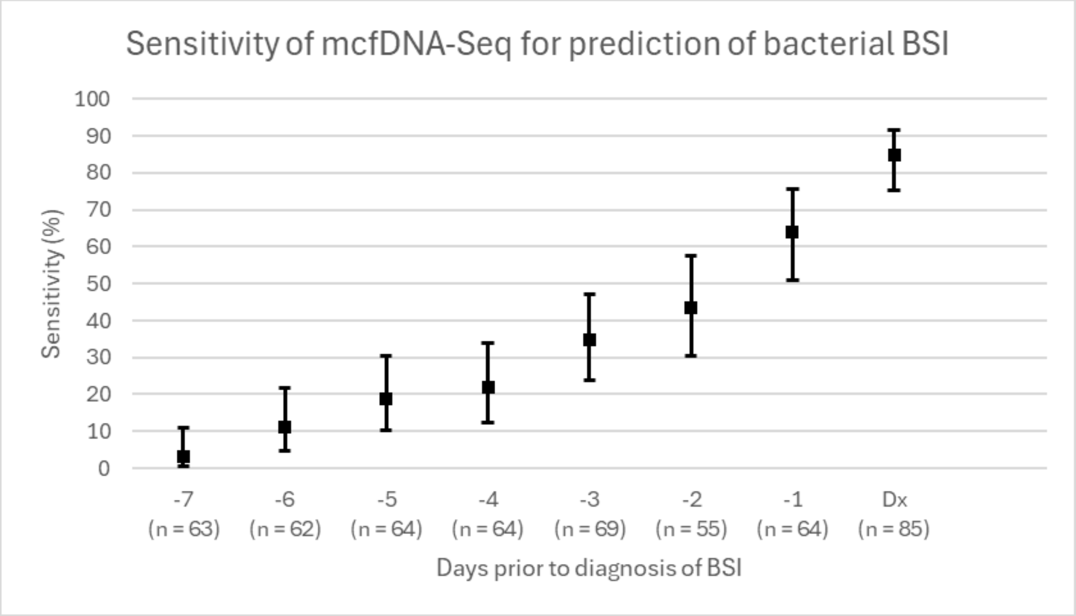

Dx, sample collected on day 0 or day +1; BSI, bloodstream infection. Figure includes consolidated data from pilot and completion phases.

eFigure 4. Sensitivity of mcfDNA-Seq for predicting bacterial bloodstream infection by days before onset (raw data)

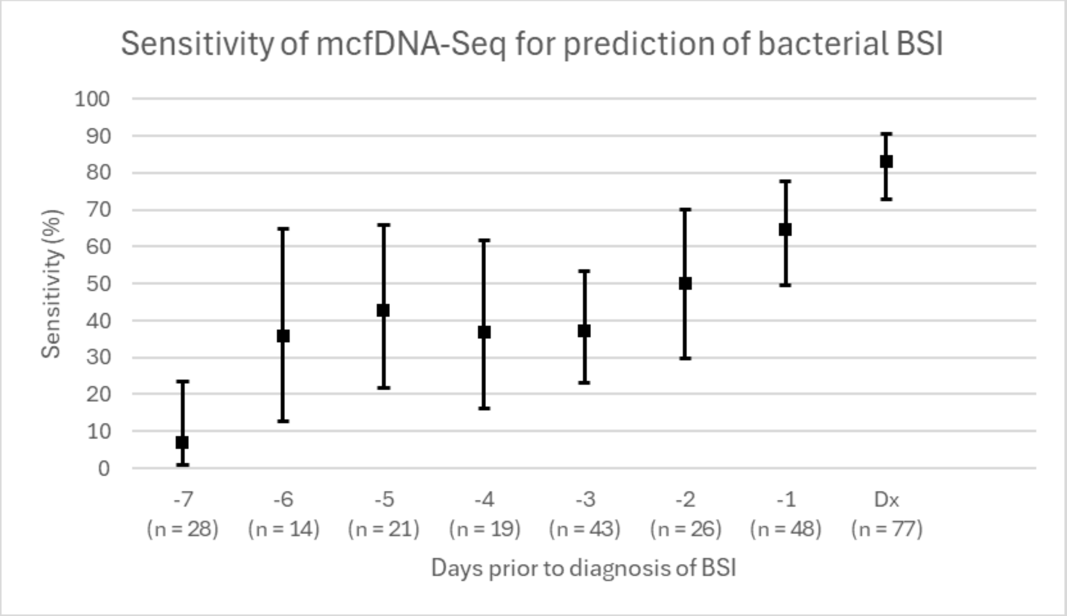

Dx, sample collected on day 0 or day +1; BSI, bloodstream infection. Figure includes consolidated data from pilot and completion phases.

eFigure 5. Violin plot of concentrations of expected microbial DNA detected by mcfDNA-Seq in BSI-related samples compared to concentrations in control samples

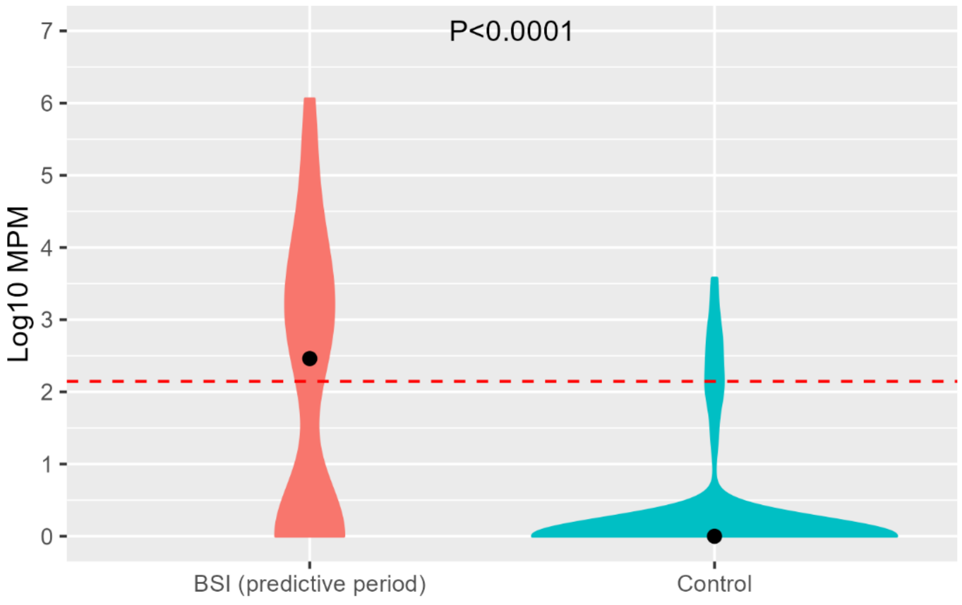

MPM, concentration of microbial DNA in molecules per microliter. Black dots represent medians for each event group; the red dashed line represents the optimal cutoff for MPM selected by the Youden index. The *P* value was derived from the Wilcoxon–Mann–Whitney test. Figure includes consolidated data from pilot and completion phases. In the predictive samples, the median log10 MPM was 2.46 (IQR 0-3.41), whereas in the control samples the median log10 MPM was 0 (IQR 0-0).

eFigure 6. Receiver operating characteristic curve for MPM values during the predictive period (Day –3 to –1) in BSI samples vs. control samples.

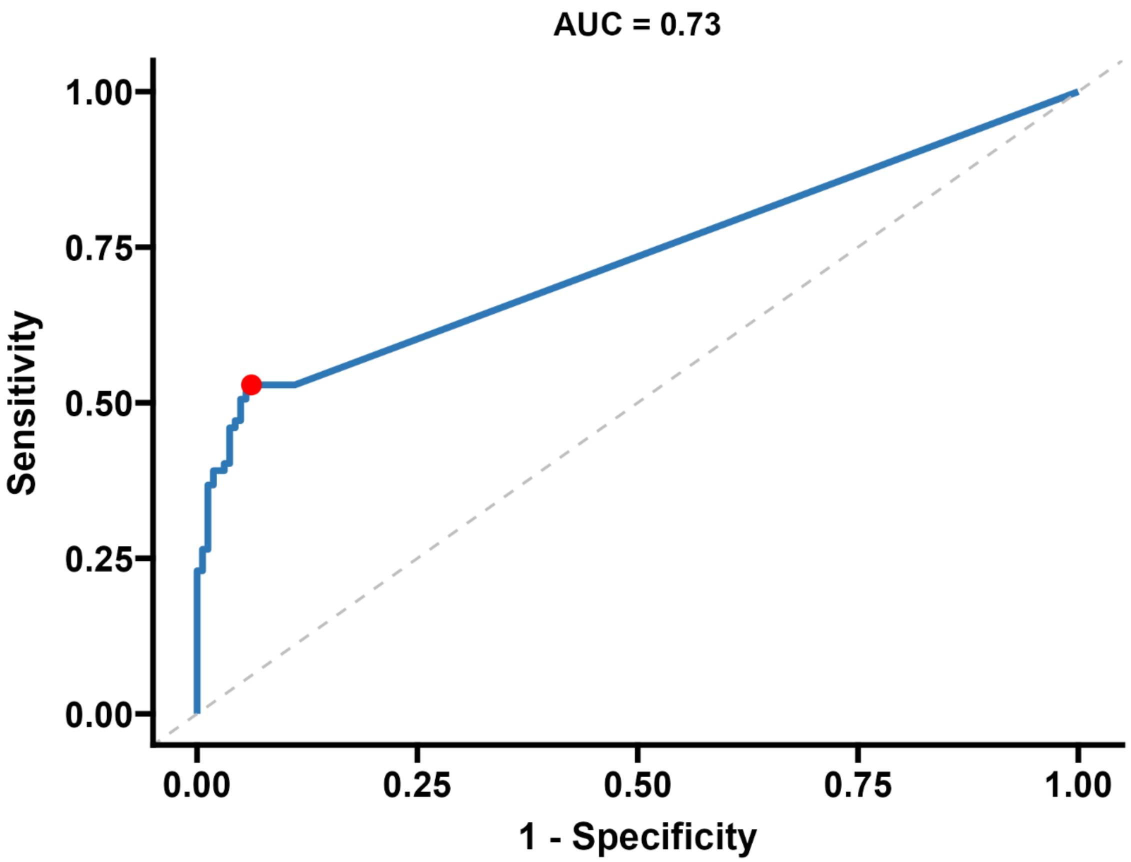

Figure shows the ROC curve based on maximum molecules per microliter (MPM) values for 87 BSI organisms and 162 control samples. The optimal cutoff, selected using the Youden index, was 140. With this cutoff, predictive sensitivity was 51.9% (95% CI: 40.5–63.1%) for all BSI episodes and predictive specificity was 90.7% (95% CI: 85.2–94.7%). Figure includes consolidated data from pilot and completion phases.

eFigure 7. Trajectory of mcfDNA-Seq–detected DNA concentrations for expected vs. additional organisms in BSI-related samples

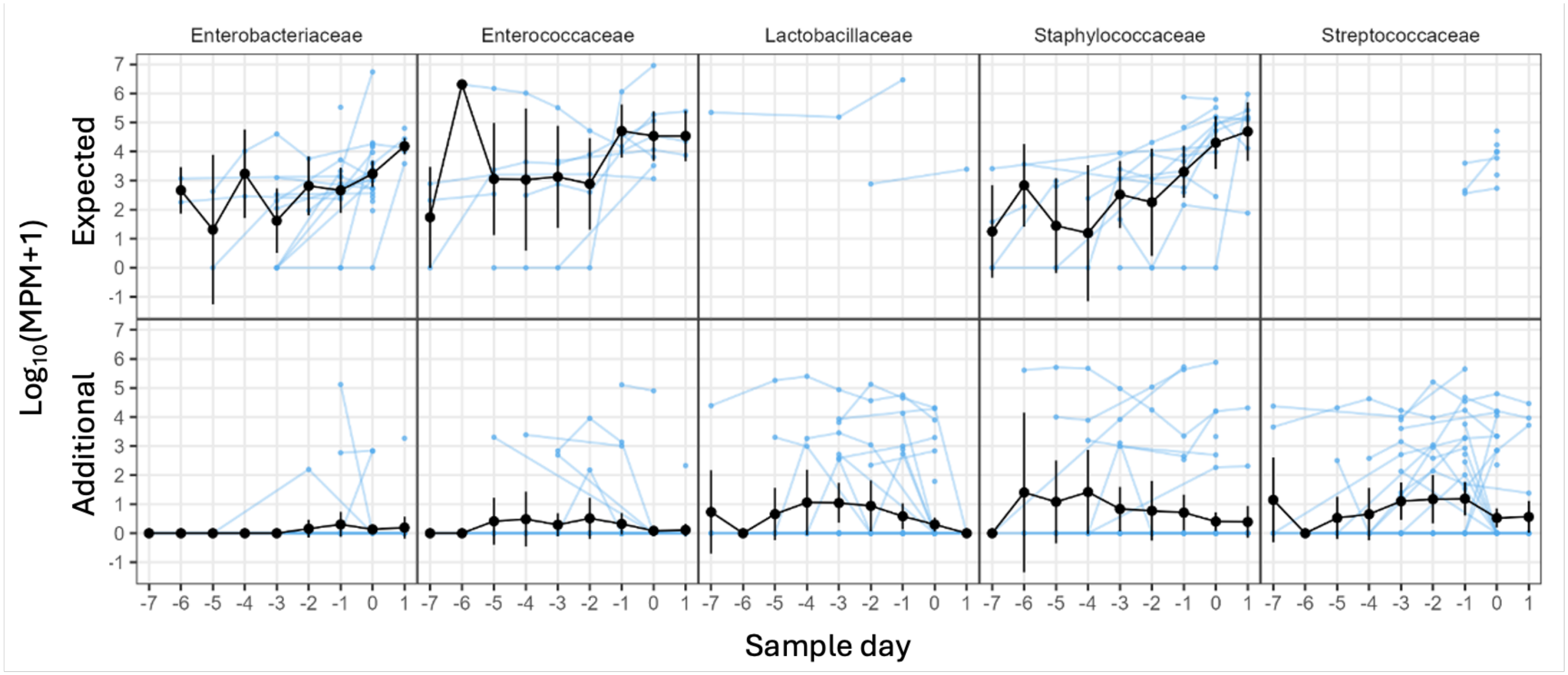

Expected organisms are defined as those identified by blood culture during the BSI episode, and additional organisms are those identified only by mcfDNA-Seq. MPM, DNA concentration in molecules per microliter of plasma. Blue dots represent observed data for each sample day, and blue lines connect samples from the same BSI episode to show trends within episodes. Black dots indicate the mean log-transformed MPM for each sample day, and error bars represent the 95% confidence intervals for the means. Means were not calculated for *Lactobacillaceae* or *Streptococcaceae* in the expected group because of the limited number of observations. Figure includes consolidated data from pilot and completion phases.

Supplemental References

1 Lehrnbecher T, Robinson PD, Ammann RA, *et al.* Guideline for the management of fever and neutropenia in pediatric patients with cancer and hematopoietic cell transplantation recipients: 2023 update. *J Clin Oncol* 2023; **41**: 1774–85.

2 Simon R. Optimal two-stage designs for phase II clinical trials. *Control Clin Trials* 1989; **10**: 1–10.

3 Goggin KP, Gonzalez-Pena V, Inaba Y, *et al.* Evaluation of plasma microbial cell-free DNA sequencing to predict bloodstream infection in pediatric patients with relapsed or refractory cancer. *JAMA Oncol* 2020; **6**: 552–6.

4 Gaur AH, Bundy DG, Werner EJ, *et al.* A prospective, holistic, multicenter approach to tracking and understanding bloodstream infections in pediatric hematology-oncology patients. *Infect Control Hosp Epidemiol* 2017; **38**: 690–6.

5 Centers for Disease Control and Prevention National Healthcare Safety network. Bloodstream Infection Event (Central Line-Associated Bloodstream Infection and Non-Central Line-Associated Bloodstream Infection). https://www.cdc.gov/nhsn/pdfs/pscmanual/4psc_clabscurrent.pdf (accessed September 25, 2025).

St. Jude
PREDSEQ
Initial version, dated: 01-20-2017, (IRB Approved: 06-23-17) Activation Date: 07-20-2017
Revision 0.1, dated 10-10-17, (IRB Approved: 10-11-2017) Activation Date: 10-11-2017
Revision 0.2, dated 04-26-2019 (IRB Approved: 5-10-2019) Activation Date: 8-5-2019
Revision 0.3, dated 10-17-2019 (IRB Approved: 10-30-2019) Activation Date: 03-16-2020
Revision 0.4, dated 07-13-2020 (IRB Approved:) Activation Date:

PREDICTION OF ADVERSE EVENTS IN CHILDREN AND ADOLESCENTS WITH CANCER AT HIGH RISK OF INFECTION (PREDSEQ)

Principal Investigator
Dr. Joshua Wolf
Department of Infectious Diseases

Co-Investigators
Dr. Li Tang[1]
Dr. Randall T. Hayden[2]
Dr. Jeffrey Rubnitz[3]
Dr. Patricia M. Flynn[4]
Dr. John Choi[2]
Dr. Gabriela Maron[4]
Dr. Elisa Margolis[4]
Dr. Jeffrey Klco[5]

[1]Department of Biostatistics
[2]Department of Pathology
[3]Department of Oncology
[4]Department of Infectious Diseases
[5]Department of Pathology

St. Jude Children's Research Hospital
262 Danny Thomas Place
Memphis, Tennessee 38105-3678
Telephone: (901) 595-3300

Contents of this document may not be extracted without permission from the Principal Investigator.

Protocol Summary

**Protocol:** PREDICTION OF ADVERSE CONSEQUENCES IN CHILDREN AND ADOLESCENTS WITH CANCER AT HIGH RISK OF INFECTION (PREDSEQ)
**Principal Investigator:** Joshua Wolf, MBBS
**IND Holder:** Not Applicable
**Brief Overview:** The majority of children and adolescents diagnosed with cancer will experience one or more episodes of fever or infection during their course of therapy. The most common microbiologically documented infection is bloodstream infection (BSI), which can be associated with severe sepsis or death.<br><br>Traditionally, diagnosis of BSI is made by culture of blood in growth media to determine whether pathogens are present. Although culture-based diagnostic techniques are important, they require a significant load of live bacteria in the blood, so in early bacteremia or focal infection preceding bacteremia, blood cultures are typically negative. The primary aim of this study is to determine whether metagenomic sequencing of cell free pathogen DNA could identify bacteremia prior to currently available clinical or laboratory approaches.<br><br>Delayed diagnosis and delayed optimal therapy of BSI is associated with increased morbidity and mortality, so novel diagnostic tools are being explored to speed up the identification and characterization of these infections. Diagnostic tools that enable rapid diagnosis of BSI on the first day of positive blood cultures are available or being explored.<br><br>However, even these rapid tests generally require active BSI to be positive, so severe sepsis may already be present before diagnosis. Ideally, a predictive test would identify patients with impending bloodstream infection to enable pre-emptive targeted therapy. Surveillance and pre-emptive therapy might be an alternative strategy to replace routine antibacterial prophylaxis. Although antibacterial prophylaxis is effective, it leads to high-density broad-spectrum antibiotic exposure and contributes to subsequent development of antibiotic resistance so a replacement would be of significant potential benefit.<br><br>One potential tool for this purpose is next generation sequencing (NGS) of pathogens to identify the presence of pathogen nucleic acids in sterile sites. In preliminary studies, NGS performed in parallel with blood culture appears to be sensitive and specific for diagnosis of BSI. However, testing prior to onset of bacteremia has not been assessed.<br><br>This study aims to determine whether next generation pathogen sequencing is able to identify the presence of pathogens in blood prior to the onset of clinical bloodstream infection.<br><br>Plasma samples collected but not required for clinical care (discarded samples) will be collected and stored. Results of NGS will be compared between patients who develop BSI immediately (within 72 hours) after sample collection, those who develop other infectious syndromes and those who remain well.

St. Jude Children's Research Hospital
IRB NUMBER: Pro00007383
IRB APPROVAL DATE: 07/29/2020

**Protocol:** PREDICTION OF ADVERSE CONSEQUENCES IN CHILDREN AND ADOLESCENTS WITH CANCER AT HIGH RISK OF INFECTION (PREDSEQ)
**Intervention:** Next generation pathogen sequencing will be performed directly on plasma samples obtained for clinical care and results immediately preceding (within 72 hours) development of bloodstream infection will be compared against others.
**Study Design:** Prospective cohort study; an exploratory phase will identify whether the test has potential utility, and a completion phase will estimate the sensitivity and specificity of the test.
**Sample Size:** Exploratory Phase, approximately 50 participants; Completion Phase, up to 200 participants.
**Data Management:** Data will be collected by staff in the Department of Infectious Diseases and maintained on a password-protected database with access limited to study staff. Only information required to perform the study will be collected.
**Human Subjects:** Because this study involves collection of personal or private information there is the risk of accidental release of this information. No other serious risks have been identified. Only samples that would otherwise be discarded will be collected.

St. Jude Children's Research Hospital
IRB NUMBER: Pro00007383
IRB APPROVAL DATE: 07/29/2020

TABLE OF CONTENTS

1.0 OBJECTIVES....1
1.1. Primary Objective....1
1.2. Secondary Objectives....1
1.3. Exploratory Objectives....1
2.0 BACKGROUND AND RATIONALE....1
2.1. Background....1
2.2. Rationale....3
2.3. Background and Rationale for Ancillary and Exploratory Studies....5
3.0 RESEARCH PARTICIPANT ELIGIBILITY CRITERIA AND STUDY ENROLLMENT....5
3.1. Inclusion Criteria....6
3.2. Exclusion Criteria (examples listed below)....6
3.3. Research Participant Recruitment and Screening....6
3.4. Enrollment on Study at St. Jude....6
3.5. Procedures for Identifying and Randomizing Research Participants....6
4.0 DESIGN AND METHODS....7
4.1. Design and Study Overview....7
5.0 REQUIRED EVALUATIONS, TESTS, AND OBSERVATIONS....8
6.0 CRITERIA FOR REMOVAL FROM PROTOCOL....8
6.1. Off Study Criteria....8
7.0 SAFETY AND ADVERSE EVENT REPORTING REQUIREMENTS....9
7.1. Reporting Adverse Experiences and Deaths to St. Jude IRB....9
8.0 DATA COLLECTION, STUDY MONITORING, AND CONFIDENTIALITY....12
8.1. Data Collection....12
8.4. Study Monitoring....12
8.5. Confidentiality....12
9.0 STATISTICAL CONSIDERATIONS....12
9.1. Anticipated Completion Dates....14
9.2. Summary of Primary and Secondary Objectives....15
10.0 OBTAINING INFORMED CONSENT....17
11.0 REFERENCES....17
APPENDIX I: EXAMPLE OF A SCHEDULE OF EVALUATIONS....18
APPENDIX III: RESEARCH TESTS....19
APPENDIX IV: SAMPLE PROCESSING....20

St. Jude Children's Research Hospital
IRB NUMBER: Pro00007383
IRB APPROVAL DATE: 07/29/2020

1.0 OBJECTIVES

1.1. Primary Objective

1.1 To estimate the sensitivity and specificity of next generation pathogen sequencing for prediction of bloodstream infection in children with cancer at high risk of infection.

1.2. Secondary Objectives

2.1 To describe the frequency and characteristics of infection episodes in a cohort of children with cancer at high risk of infection.

2.2 To estimate the sensitivity and specificity of next generation pathogen sequencing for prediction of any microbiologically documented infection in children with cancer at high risk of infection.

1.3. Exploratory Objectives

3.1 To explore the results of next generation pathogen sequencing during clinically or microbiologically documented infection or febrile neutropenia in children with cancer at high risk of infection.

3.2 To explore other alternative approaches to prediction, diagnosis or severity assessment for infectious syndromes in children with cancer at high risk of infection.

3.3 To use excess plasma from these studies to explore the frequency of somatic mutations in cell-free DNA from children with relapsed leukemia and correlate these with peripheral blood blast count and minimal residual disease levels.

2.0 BACKGROUND AND RATIONALE

2.1. Background

BSI is the most common microbiologically documented infectious complication of therapy for leukemia in children. [Fratino, 2005; Inaba, 2017] Microbial contamination of a central venous catheter (CVC) at the time of insertion, or during use, can lead to accumulation of organisms on the catheter, predominantly on the luminal surface, and eventually to development of symptomatic BSI. [Raad, 1993] Further, mucositis from cytotoxic chemotherapy can allow

St. Jude Children's Research Hospital
IRB NUMBER: Pro00007383
IRB APPROVAL DATE: 07/29/2020

translocation of bacteria across the oral or gastrointestinal mucosa. [Flagg, 2015; Epstein, 2016]

BSI Rates

Rates of BSI vary between institutions, CVC types and patient groups, but the problem does not appear to be eliminable. Recent studies in pediatric cancer patients show a rate of 1.6 – 2.8 BSI episodes per 1000 line days. [Allen, 2008; Henrickson, 2000; Hord, 2011] However, some patients are at much higher risk. Factors contributing to risk include profound neutropenia, external CVC, mucosal injury, bone marrow transplantation and tumor type. Patients with leukemia or relapsed malignancies are at highest risk. [Ammann, 2015] Further, organisms differ between patient populations, cancer therapy and prophylaxis regimens. Patients with relapsed leukemia are at especially high risk of viridans group streptococcal infection. [Nielsen, Med, 2015]

A number of groups of St. Jude patients have these high-risk features, including those with medulloblastoma or neuroblastoma, acute myeloid leukemia, relapsed leukemia and following bone marrow transplantation. For example, in patients with relapsed leukemia undergoing therapy for relapsed leukemia on the RELHEM, RELHEM2 and SELHEM trials, bacterial bloodstream infection was identified in 15 of 77 (19.5%) participants during their first course of experimental therapy. The initial recruitment will focus on patients with relapsed or refractory leukemia, and other populations will be accessed as needed to ensure adequate recruitment.

Mortality, Sepsis and Clinical Complications

In the pediatric oncology population, mortality attributable to BSI is rare (~2% of episodes), [Adler, 2006] but clinical sepsis occurs in 9 - 19% of episodes [Adler, 2006; Flynn, 2003; Aledo, 1998], and many patients require intensive care unit (ICU) admission. Many BSIs are related to central venous catheters, and the rate and proportion of catheter loss due to infection varies considerably between studies, depending on local salvage protocols and duration of follow-up, however rates of up to 37 - 46% are reported. [Stamou, 1999; Adler, 2006] During induction therapy for acute lymphoblastic leukemia, even patients with non-fatal bacteremia frequently have evidence of severe infection. Analysis of severity and complications of non-fatal bloodstream infections in children undergoing chemotherapy for acute lymphoblastic leukemia found that severe disease was common. [Wolf, Unpublished] Of 154 bacterial bloodstream infections identified in these participants, 34 (22.1%) had severe sepsis or septic shock, or required supplemental oxygen, fluid bolus or intensive care. Further, the risk of severe disease was highest during the reintensification phases (4/7 events, 57.1%), which most closely reflect the population that will be studied in this trial.

St. Jude Children's Research Hospital
IRB NUMBER: Pro00007383
IRB APPROVAL DATE: 07/29/2020

Length of Hospital Stay and Attributable Cost

In addition to clinical complications, the resource and financial costs of BSI in pediatric oncology patients is high. One study in pediatric oncology patients found that central-line related BSI was associated with a mean attributable cost of $69,332. [Wilson, 2014] The same study found that the mean attributable increase in length of stay was 21.2 days. This is consistent with another study of central-line related BSI in pediatric patients which did not focus on the oncology population. [Goudie, 2014] This study found a mean attributable length of stay of 19 days and cost of $55,646. Bloodstream infection is also the most expensive hospital acquired infection in adults, according to a large 2012 study, with a mean cost of $45,814. [Zimlichman, 2013]

2.2. Rationale

The most important modifiable risk factor for clinical complications and mortality in patients with sepsis is delay in administration of antibiotics. The odds of mortality from bloodstream infection rise with delay of appropriate antibiotic therapy. [Lodise, 2007] This is especially true in profoundly neutropenic patients, in whom a 24 hour delay in appropriate therapy for bloodstream infection was associated with an adjusted odds ratio of 17.2 for mortality, compared to an adjusted odds ratio of 1.75 for non-neutropenic patients. [Lin, 2008] A one hour delay in antibiotic administration for severe sepsis is associated with a 46% increase in the odds of mortality. [Sterling, 2015] An effective predictive test could eliminate delay in initiation of antibiotics by identification of patients before the onset of high-grade bacteremia and allowing pre-emptive treatment.

This study uses next generation pathogen sequencing to identify cell-free DNA in plasma samples collected as part of clinical care from children at high risk of infection. Next generation metagenomic sequencing (NGS) amplifies all available strands of pathogen genome to allow a more unbiased approach than competing technologies such as 16s rRNA or other PCR techniques. NGS from blood in patients with leukemia, using a process similar to that used in this study, has been shown to differentiate patients with fever from those without, but has not been used to diagnose undifferentiated bloodstream infection. [Gyarmati, 2016] Further, a similar technique was used to identify invasive astrovirus infection in an immunocompromised patient with encephalitis. [Naccache, 2015]

The NGS technique that will be used in this study was developed by Karius inc. by optimizing previously published methods for clinical use. [De Vlaminck, 2013, De Vlaminck 2015]. The procedure uses several parallel techniques to enrich pathogen DNA at the preparation, sequencing and bioinformatic steps of the process, allowing computational power to be focused on pathogen identification. The method has been tested for correlation with results of blood cultures and

St. Jude Children's Research Hospital
IRB NUMBER: Pro00007383
IRB APPROVAL DATE: 07/29/2020

found to have reasonable sensitivity and specificity. One study of NGS using the Karius platform showed that 80.0% of positive blood cultures were identified in samples collected on the same day. [Hong, 2016]. Specificity of the test in this setting was good (73.8%), and in patients with negative blood cultures but positive NGS testing, the same organism was often (26.3% of cases) identified from another site suggesting that 'false positives' are clinically relevant. Accounting for all culture-based information (including blood, respiratory tract, intraperitoneal and urinary samples) the positive and negative agreement for plasma NGS were 82.4% and 79.1%. Additional clinical information was not available to determine whether the other 'false negatives' were clinically consistent.

Table 1. Performance of the plasma-based next-generation sequencing assay in patients with suspected bacteremia [Hong, 2016]

	Blood culture		Any culture-based test	
	+	-	+	-
Plasma NGS				
+	100	38	112	28
-	25	107	24	106

A number of clinical studies are underway to assess the performance of this assay in adult patients with cancer (NCT02912117) or undergoing hematopoietic stem cell transplantation (NCT02804464). These studies will all assess performance at the time of clinical or laboratory diagnosis of infection rather than prior to its onset.

The use of blood samples that are collected for clinical care but not required allows the study to obtain samples prior to the onset of a clinical event at regular intervals. Frequent sampling for research purposes would be an inappropriate burden, and less regular sampling would likely miss the 72 hour window period for many BSI events. However, because patients undergoing therapy for cancer have frequent collection of CBC for monitoring of hemoglobin, platelets and leukocyte counts and the majority of the sample is unused (Gawad C, personal communication) there is sufficient leftover sample on most days for testing. On average, eligible patients are expected to have blood collected on 70% of days, so missing a 72 hour window prior to a BSI event is expected to be relatively uncommon.

Bloodstream infection was chosen as the primary endpoint because it is the most common microbiologically proven infection in children with cancer, and because of the clinical ramifications described in the Background. [Inaba, 2017] Further, the sensitivity of the test might be highest for BSI because there are live bacteria in the blood at the time of diagnosis. However, because the test examines cell-free

St. Jude Children's Research Hospital
IRB NUMBER: Pro00007383
IRB APPROVAL DATE: 07/29/2020

DNA, the effect of the presence of bacterial cells in the bloodstream on the sensitivity of the test is unknown.

As the performance of the test for this purpose is unknown, the exploratory phase will assess the alternate hypothesis that sensitivity is below the clinically useful range. Antibacterial prophylaxis typically reduces the risk of infection by around 50%, but risks are high. If this new test did not predict at least 30% of bloodstream infections to allow pre-emptive therapy, it would be unlikely to have a significant impact on clinical care, and we would like the test to be able to detect at least 50% of bloodstream infections. Therefore, we aim to determine whether the sensitivity of the test is within this clinically useful range. A two-stage Simon's design was employed [Simon, 1989]

2.3. Background and Rationale for Ancillary and Exploratory Studies

A clinical description of infectious complications in a broad group of patients with relapsed leukemia is an important potential contribution. Although many studies have reported that relapsed leukemia is an important risk factor for serious infection, [Lai, 2003; Hale, 2010] there are few available data for risk stratification and determination of risk factors for infection. A Pubmed search on 11-22-16 using the search strategy "infection relapsed leukemia (Pediatric OR paediatric OR child OR infant)", with exclusion of irrelevant, adult-only publications, HSCT-related publications and case reports, identified a number of individual trial results, but no multi-trial prospective studies aimed at identification of risk factors for serious infection in this population. The studies all showed that patients undergoing therapy for relapsed leukemia were at high risk of infection. [Ochs, 1990; Wells, 1994; Leblanc, 1994; Lockhart, 1994; Wells, 2003; Morland, 1996; Whitlock, 1997; McCarthy, 1999; Thomson, 2004; Berg, 2011; Lawson, 2000; Hijiya, 2008; Jeha, 2009; Hijiya, 2011; Messinger, 2012; Trioche, 2012] However, additional risk factors or time-periods were not reported. This study will provide information about absolute risk, risk reduction and risk stratification that can be used for clinical care and future research.

Estimation of the sensitivity and specificity of NGS for prediction of any infectious episodes in children with malignancies at high risk of infection will further assist in the determination of a possible clinical role for this test. Although some episodes of infection are associated with bloodstream infection, many are clinically or microbiologically diagnosed infection at a specific site, or fever without a clear source or etiology. In a study of children with acute lymphoblastic leukemia undergoing induction therapy, only 14.4% of episodes were associated with bloodstream infection, whereas 38.7% were other documented infections, and 48.0% were febrile neutropenia without a documented source. [Inaba, 2017] Therefore, the specificity of the test may appear very poor if accounting only for BSI. The potential value of this secondary analysis is to determine the maximum specificity of the test for infection-related episodes.

St. Jude Children's Research Hospital
IRB NUMBER: Pro00007383
IRB APPROVAL DATE: 07/29/2020

Circulating tumor DNA holds great promise as a noninvasive method for detecting and monitoring the treatment responses of malignancies [Bettegowda, 2014] Current flow cytometric and immune receptor-based methods for monitoring minimal residual disease have suboptimal sensitivity and specificity. As high-risk and relapse leukemia patients are more likely to have high levels of residual disease during treatment, we will use this exploratory objective as a first measure of how the frequency of somatic variants in cell-free DNA correlate with standard minimal residual disease measurements and peripheral blast count. After 50 patients, if the exploratory studies provide data to support a larger study, a separate protocol will be submitted.

3.0 RESEARCH PARTICIPANT ELIGIBILITY CRITERIA AND STUDY ENROLLMENT

According to institutional and NIH policy, the study will accession research participants regardless of gender and ethnic background. Institutional experience confirms broad representation in this regard.

3.1. Inclusion Criteria

3.1.1. Under 25 years of age at time of study enrollment

3.1.2. Undergoing care for cancer at St. Jude

3.1.3. In a category of patients who are considered by the investigator to be at high risk of infection

3.1.4. Expected to receive care at St. Jude for at least 7 days

3.2. Exclusion Criteria

3.2.1. Any condition that would, in the opinion of the investigator, place the subject at an unacceptable risk of injury or render the subject unable to meet the requirements of the protocol

3.3. Research Participant Recruitment and Screening

Participants will be recruited from St. Jude Children's Research Hospital. Potential participants will be identified by direct contact with primary clinicians, from acceptance and enrollment notices for new patients.

Based on data from 2015 – 16, it is expected that around 20 participants will be eligible for inclusion annually.

St. Jude Children's Research Hospital
IRB NUMBER: Pro00007383
IRB APPROVAL DATE: 07/29/2020

3.4. Enrollment on Study at St. Jude

A member of the study team will confirm potential participant eligibility as defined in Section 3.1-3.2, complete and sign the 'Participant Eligibility Checklist'. The study team will enter the eligibility checklist information into the Patient Protocol Manager (PPM) system, and sign the completed checklist.

The CTO is staffed 7:30 am-5:00 pm CST, Monday through Friday. A staff member from the Milli helpline is on call Saturday, Sunday, and holidays from 8:00 am to 6:00 pm. If you have a prospective research enrollment and need assistance releasing your consent, please call the Milli helpline (901-338-0596) on call number.

3.5. Procedures for Identifying Research Participants

Potential study participants will be identified from contact with primary clinicians, protocol enrollment records and institution acceptance records. A request for waiver of all elements of consent will be made because of the minimal risk nature of the protocol (leftover specimens and existing clinical data only) and the active COVID-19 pandemic. Because of the observational nature of the trial re-consent will not be obtained at age of majority in children and adolescents.

4.0 DESIGN AND METHODS

4.1. Design and Study Overview

This pilot study is a single arm observational cohort study. Clinical data describing baseline information about the patient and malignancy, antibiotic and chemotherapy exposure, microbiology testing, hematology results and infection-related events will be collected prospectively from the electronic medical record, pharmacy and laboratory databases into a password protected database maintained by the Department of Infectious Diseases according to usual departmental protocol.

Leftover clinical blood samples collected in EDTA will be collected up to daily from the clinical hematology laboratory and processed using standard plasma collection procedures.

The blood will be spun at low speed for separation using Ficoll-Paque PLUS. This will be followed by transferring the plasma to a new tube and a high-speed spin to pellet any remaining cells. The supernatant that contains the plasma and cell-free DNA will then be transferred into aliquots that contain a maximum of 750ul prior to freezing at -80 degrees Celsius. The cells from the mononuclear

St. Jude Children's Research Hospital
IRB NUMBER: Pro00007383
IRB APPROVAL DATE: 07/29/2020

cell layer will also be removed, resuspended in freezing media, and frozen in liquid nitrogen.

After identification of relevant episodes, plasma samples will be transferred to Karius for testing by sending a 750ul aliquot for each patient to be tested on dry ice. The samples will be blinded during testing to reduce the risk of bias in interpretation.

A group of samples that contain greater than 750ul of plasma or are determined by the PI not to be required for the primary infectious aims of the study will undergo standard cell-free DNA isolation, followed by library preparation and exome sequencing to identify persistent circulating somatic mutations. This will only be performed for participants who have provided prior authorization for genomic sequencing in a research context and have already undergone germline and tumor sequencing by Clinical Genomics. Thus, it is not expected that previously unidentified germline variants, necessitating reporting to participants, would be identified. Any such variants would have already been identified through sequencing performed in a CLIA setting through the Clinical Genomics Laboratory. The clinical significance of new somatic variants identified by circulating tumor DNA during treatment has not been established, so somatic variants that were not found in the diagnostic clinical sample would not be reported to participants. The allele frequency of those mutations will be correlated with persistent circulating blasts in the patient's blood and minimal residual disease levels.

We will apply Simon’s two-stage design for the primary objective. Our minimal acceptable sensitivity is 30% and favorable sensitivity is 50%. The exploratory stage is designed to ensure early trial discontinuation if the sensitivity of the test is unlikely to reach 50%. Setting the minimal acceptable sensitivity at 30% provides a 72% probability of early trial discontinuation if the true sensitivity is lower than 30%.

In this exploratory stage, we will obtain 15 BSI episodes, and if 5 or fewer correct positive diagnoses are made by NGS, we would stop the study early for futility. The completion stage is designed initially (for the first 46 participants, including the 15 from the exploratory stage) to assess whether the sensitivity of the test is greater than 50%, and then subsequently to precisely estimate the sensitivity and specificity of the test. The value of 50% as the favorable sensitivity was chosen because it represents the approximate efficacy of antibacterial prophylaxis, which has been implemented in this population. [Gafter-Gvili, 2012]

During the completion stage, we will aim to achieve a total of 100 BSI episodes. If out of the first 46 included events, NGS correctly diagnoses 18 or fewer positive cultures, the NGS test does not achieve satisfactory sensitivity performance and would not warrant further investigation for this purpose. The

St. Jude Children's Research Hospital
IRB NUMBER: Pro00007383
IRB APPROVAL DATE: 07/29/2020

overall target sample size is 100 BSI events with the aim of estimating the sensitivity and specificity of the test to a 95% confidence interval of ±10%, assuming a sensitivity of ~50% and specificity of 80%. Samples that are not processed to plasma and frozen within the first 100 hours after collection (Delayed Processing) will be reported separately as the delay in processing might affect the sensitivity or specificity of the test. Therefore, events for which all predictive samples have Delayed Processing will not be included in the denominator for evaluation of test sensitivity, but may be reported to describe the effect of delays in sample processing on test utility.

Data collection forms for infection-related complications are provided in Appendix 2.

4.2. Human Genetic Studies

Prior to cell-free DNA (cfDNA) isolation, we will examine each patient's clinical chart to insure they have been consented by the Tissue Bank, and have already consented to undergo Clinical Sequencing.

Cell-free exomes may be compared to the germline exome from the same patient using the germline exome BAM file, which will be requested through the Sequencing Data Access Committee. The standard somatic variant calling pipeline does not identify variants that are present in both the leukemia cells and germline sample. Consequently, we will not examine germline variants. It is possible we will identify somatic variants that were selected for during treatment. However, the clinical significance of those variants are not known.

4.3. Return of Incidental and Secondary Genetic Findings:

Because testing for pathogens and somatic mutations will be batched and is likely to be delayed beyond clinical usefulness, and because cell-free DNA testing has not been shown to be clinically predictive of infection or cancer outcomes in this setting, there is no plan to return the results of cell-free testing for either pathogens or somatic mutations. Further, as a result of the variant calling pipeline and because only participants who have undergone germline sequencing will be eligible for somatic mutation identification, previously unidentified germline mutations will not be discovered. The clinical value of somatic mutations identified by cfDNA sequencing that had not been identified at diagnosis is unknown, so they will not be returned.

However, in the unforeseen event that a potentially clinically significant genetic finding was identified, the principal investigator would present the finding to the Institutional Review Board and invite guidance.

St. Jude Children's Research Hospital
IRB NUMBER: Pro00007383
IRB APPROVAL DATE: 07/29/2020

5.0 REQUIRED EVALUATIONS, TESTS, AND OBSERVATIONS

Data and plasma samples will be collected throughout the study.

Data to be collected:

- Baseline information about demographics, past infection-related episodes, primary disease and past treatment will be collected at the time of enrollment.
- Data about antimicrobial use, including drug, route and indication will be collected for 30 days prior to study enrollment and then throughout the study.
- Data about infection-related complications, including febrile neutropenia and clinically or microbiologically documented infection, and cancer outcomes will be collected for 30 days prior to study enrollment, then throughout the study.

Plasma samples will be collected directly from the clinical pathology laboratories when leftover blood is available.

Samples to be collected:

- Plasma samples will be collected daily throughout the study if leftover blood is available. No samples will be collected specifically for the study.

6.0 CRITERIA FOR REMOVAL FROM PROTOCOL

6.1. Off Study Criteria

6.1.1. All protocol interventions are complete
6.1.2. Death
6.1.3. Lost to follow-up or no longer receiving care at St. Jude
6.1.4. Request of the Patient/Parent
6.1.5. Patient no longer requires treatment for cancer
6.1.6. Discretion of the Study PI, such as the following:

- The researcher decides that continuing in the study would be harmful
- Blood testing is not expected to be performed at least weekly for clinical care

7.0 SAFETY AND ADVERSE EVENT REPORTING REQUIREMENTS

7.1. Reporting Adverse Experiences and Deaths to St. Jude IRB

7.1.1. Only "unanticipated problems involving risks to participants or others" referred to hereafter as "unanticipated problems" are required to be reported to the St. Jude IRB promptly, but in no event later than 10 working days after the investigator first learns of the unanticipated problem. Regardless of whether the event is internal or external (for example, an IND safety report by the sponsor pursuant to 21 CFR

St. Jude Children's Research Hospital
IRB NUMBER: Pro00007383
IRB APPROVAL DATE: 07/29/2020

312.32), only adverse events that constitute unanticipated problems are reportable to the St. Jude IRB. As further described in the definition of unanticipated problem, this includes any event that in the PI's opinion was:

- Unexpected (in terms of nature, severity, or frequency) given (1) the research procedures that are described in the protocol-related documents, such as the IRB-approved research protocol and informed consent document, as well as other relevant information available about the research; (2) the observed rate of occurrence (compared to a credible baseline for comparison); and (3) the characteristics of the subject population being studied; and
- Related or possibly related to participation in the research; and
- Serious; or if not serious suggests that the research places subjects or others at a greater risk of harm (including physical, psychological, economic, or social harm) than was previously known or recognized.

Unrelated, expected deaths do not require reporting to the IRB. Though death is "serious", the event must meet the other two requirements of "related or possibly related" and "unexpected/unanticipated" to be considered reportable.
Deaths meeting reporting requirements are to be reported immediately to the St. Jude IRB, but in no event later than 48 hours after the investigator first learns of the death.

7.1.2. The following definitions apply with respect to reporting adverse experiences:

7.1.2.1. **Serious Adverse Event**: Any adverse event temporally associated with the subject's participation in research that meets any of the following criteria:

- results in death;
- is life-threatening (places the subject at immediate risk of death from the event as it occurred);
- requires inpatient hospitalization or prolongation of existing hospitalization;
- results in a persistent or significant disability/incapacity;
- results in a congenital anomaly/birth defect; or
- any other adverse event that, based upon appropriate medical judgment, may jeopardize the subject's health and may require medical or surgical intervention to prevent one of the other outcomes listed in this definition (examples of such events include: any substantial disruption of the ability to conduct normal life functions, allergic bronchospasm requiring intensive treatment in the emergency room or at home, blood dyscrasias or convulsions that do not result in inpatient hospitalization, or the development of drug dependency or drug abuse), a

St. Jude Children's Research Hospital
IRB NUMBER: Pro00007383
IRB APPROVAL DATE: 07/29/2020

congenital anomaly/birth defect, secondary or concurrent cancer, medication overdose, or is any medical event which requires treatment to prevent any of the medical outcomes previously listed.

7.1.2.2. Unexpected Adverse Event:

- Any adverse event for which the specificity or severity is not consistent with the protocol-related documents, including the applicable investigator brochure, IRB approved consent form, Investigational New Drug (IND) or Investigational Device Exemption (IDE) application, or other relevant sources of information, such as product labeling and package inserts; or if it does appear in such documents, an event in which the specificity, severity or duration is not consistent with the risk information included therein; or
- The observed rate of occurrence is a clinically significant increase in the expected rate (based on a credible baseline rate for comparison); or
- The occurrence is not consistent with the expected natural progression of any underlying disease, disorder, or condition of the subject(s) experiencing the adverse event and the subject's predisposing risk factor profile for the adverse event.

7.1.2.3. **Internal Events**: Events experienced by a research participant enrolled at a site under the jurisdiction of St. Jude IRB for either multicenter or single-center research projects.

7.1.2.4. **External Events:** Events experienced by participants enrolled at a site external to the jurisdiction of the St. Jude Institutional Review Board (IRB) or in a study for which St. Jude is not the coordinating center or the IRB of record.

7.1.2.5. **Unanticipated Problem Involving Risks to Subjects or Others:** An <u>unanticipated problem involving risks to subjects or others</u> is an event which was not expected to occur and which increases the degree of risk posed to research participants. Such events, in general, meet all of the following criteria:

- unexpected;
- related or possibly related to participation in the research; and
- suggests that the research places subjects or others at a greater risk of harm (including physical, psychological, economic, or social harm) than was previously known or recognized. An

St. Jude Children's Research Hospital
IRB NUMBER: Pro00007383
IRB APPROVAL DATE: 07/29/2020

unanticipated problem involving risk to subjects or others may exist even when actual harm does not occur to any participant.

7.1.3. Consistent with FDA and OHRP guidance on reporting unanticipated problems and adverse events to IRBs, the St. Jude IRB does not require the submission of external events, for example IND safety reports, nor is a summary of such events/reports required; however, if an event giving rise to an IND safety or other external event report constitutes an "unanticipated problem involving risks to subjects or others" it must be reported in accordance with this policy. In general, to be reportable external events need to have implications for the conduct of the study (for example, requiring a significant and usually safety-related change in the protocol and/or informed consent form).

7.1.4. Although some adverse events will qualify as unanticipated problems involving risks to subjects or others, some will not; and there may be other unanticipated problems that go beyond the definitions of serious and/or unexpected adverse events. Examples of unanticipated problems involving risks to subjects or others include:

- Improperly staging a participant's tumor resulting in the participant being assigned to an incorrect arm of the research study;
- The theft of a research computer containing confidential subject information (breach of confidentiality); and
- The contamination of a study drug.
  Unanticipated problems generally will warrant consideration of substantive changes in the research protocol or informed consent process/document or other corrective actions in order to protect the safety, welfare, or rights of subjects or others.

8.0 DATA COLLECTION, STUDY MONITORING, AND CONFIDENTIALITY

8.1. Data Collection

Clinical data will be collected by study staff from the Department of Infectious Diseases from the electronic medical record and from protocol, pharmacy, laboratory and other institutional databases. Data will be collected on paper case report forms, and entered into a password-protected study database. The study database will be accessible only to study staff.

8.2. Study Monitoring

The Principal Investigator and study team are responsible for ensuring protocol compliance. 100% of eligibility checklists will be reviewed for accuracy and

completeness by the Eligibility Coordinators. Monitoring of timeliness of serious adverse event reporting will be done as events are reported in TRACKS.

8.3. Confidentiality

Protected health information will be obtained from participants as part of this study, including medical record number, date of birth, and details of clinical care. To protect participants, the research database and any data collection forms containing protected health information will be maintained in a locked room or file-cabinet or in a password protected file. No research participant names will be recorded on data collection forms. A unique patient identifier will be used instead of the medical record number in all other documentation. The list containing the link between study number and medical record number will be maintained in a locked file. All potentially identifying participant information will be accessible only by study staff. The medical records of study participants may be reviewed by the St. Jude IRB, FDA, clinical research monitors, etc.

8.4. Data Deposition

Anonymized cfDNA exome data (BAM or FASTQ files) will be deposited in accordance with practices established by the St. Jude Pediatric Cancer Genome Project and in accordance with the NIH Genomic Data Sharing (GDS) policy. Human exome data that would have only been generated from patients with a Tissue Bank consent will be deposited and maintained in the Short Read Archive at NCBI. To facilitate data sharing and because exome data is not identifiable, we will not place it in a database with more restricted access, such as dbGaP.

9.0 STATISTICAL CONSIDERATIONS

Sample size considerations are outlined in Section 4.1.

9.1. Primary Objective

To estimate the sensitivity and specificity of next generation pathogen sequencing for prediction of bloodstream infection in children with cancer at high risk of infection.

Sensitivity is defined as the proportion of NGS positive results in all positive BSI cultures. Specificity is defined as the proportion as NGS negative results in all negative BSI cultures. Proportions and 95% confidence intervals will be reported.

9.2. Secondary Objectives

St. Jude Children's Research Hospital
IRB NUMBER: Pro00007383
IRB APPROVAL DATE: 07/29/2020

9.2.1 To describe the frequency and characteristics of infection episodes in a cohort of children with cancer at high risk of infection.

Frequency of infection episodes will be summarized. Characteristics of those episodes will be reported with appropriate descriptive statistics (frequency and proportion for categorical variables and mean/median etc. for continuous variables) and risk factors for infection identified by univariate and multivariate testing as appropriate.

9.2.2 To estimate the sensitivity and specificity of next generation pathogen sequencing for prediction of any microbiologically documented infection in children with cancer at high risk of infection.

Sensitivity and specificity of NGS predicting any microbiologically documented infections will be reported as proportions along with 95% confidence intervals.

9.3. Exploratory Objectives

9.3.1 To explore the results of next generation pathogen sequencing during clinically or microbiologically documented infection or febrile neutropenia in children with cancer at high risk of infection.

Sensitivity and specificity of NGS predicting any clinically or microbiologically documented infections or febrile neutropenia will be reported as proportions along with 95% confidence intervals.

9.3.2 To explore other alternative approaches to prediction, diagnosis or severity assessment for infectious syndromes in children with cancer at high risk of infection.

Sensitivity and specificity of alternative approaches predicting, diagnosing or assessing severity for infectious syndromes will be reported as proportions along with 95% confidence intervals. Multivariate logistic regression may be explored to assess the impact of identified risk factors on prediction, diagnosis or severity of infectious syndromes.

9.3.3 To explore the frequency of somatic mutations in cell free DNA from children with high-risk leukemia and correlate these with peripheral blood blast count and minimal residual disease levels.

Frequency of somatic mutations will be summarized and correlation coefficient will be reported with peripheral blood blast count and minimal residual disease levels.

9.1. Anticipated Completion Dates

**Anticipated Primary Completion Date:** December 31, 2020

**Anticipated Study Completion Date**: December 31, 2021

St. Jude Children's Research Hospital
IRB NUMBER: Pro00007383
IRB APPROVAL DATE: 07/29/2020

9.2. Summary of Primary and Secondary Objectives

Primary and Secondary Summary of Objectives

Objective #	Objective Type	Analysis #	Resp Party	Stat	Safety	Analysis Measure	Analysis Title	Data Collection Time Frame	# of Participants
1.1	P	3	JW	LT	N	Episode	Estimate the sensitivity and specificity of next generation pathogen sequencing for BSI	Throughout study	Up to 100
2.1	S	1	JW	LT	N	Episode	Describe frequency and characteristics of infection	Throughout study	Up to 200
2.2	S	1	JW	LT	N	Episode	Estimate sensitivity and specificity of next generation pathogen sequencing for prediction of any infection	Throughout study	Up to 200
3.1	S	1	JW	LT	N	Episode	Explore results of next generation pathogen sequencing during documented infection	Throughout study	Up to 200
3.2	S	As needed	JW	LT	N	Episode	Explore alternative approaches to prediction, diagnosis or severity assessment for infection	Throughout study	Up to 200
3.3	S	As needed	CG	LT	N	Episode	Explore frequency of resistance mutations in cell free DNA from children with high-risk leukemia	Throughout study	Up to 200

St. Jude Children's Research Hospital
IRB NUMBER: Pro00007383
IRB APPROVAL DATE: 07/29/2020

10.0 OBTAINING INFORMED CONSENT

Potential study participants will be identified from contact with primary clinicians, protocol enrollment records and institution acceptance records. A request for waiver of all elements of consent will be made because of the minimal risk nature of the protocol (leftover specimens and existing clinical data only) and the active COVID-19 pandemic.

All research participants who meet eligibility criteria regardless of gender or minority status are fully eligible to participate in this study. All data will be kept confidential and stored in locked offices.

10.1 Consent When English is Not the Primary Language

Potential study participants will be identified from contact with primary clinicians, protocol enrollment records and institution acceptance records. A request for waiver of all elements of consent will be made because of the minimal risk nature of the protocol (leftover specimens and existing clinical data only) and the active COVID-19 pandemic.

St. Jude Children's Research Hospital
IRB NUMBER: Pro00007383
IRB APPROVAL DATE: 07/29/2020

11.0 REFERENCES

1. A. Adler *et al.*, Catheter-associated bloodstream infections in pediatric hematology-oncology patients: factors associated with catheter removal and recurrence. *J. Pediatr. Hematol. Oncol.* **28**, 23 (Jan, 2006).
2. A. Aledo *et al.*, Septicemia and septic shock in pediatric patients: 140 consecutive cases on a pediatric hematology-oncology service. *J. Pediatr. Hematol. Oncol.* **20**, 215 (May-Jun, 1998).
3. R. C. Allen *et al.*, Risk determinants for catheter-associated blood stream infections in children and young adults with cancer. *Pediatr. Blood Cancer* **51**, 53 (Jul, 2008).
4. R. A. Ammann *et al.*, Bloodstream infection in paediatric cancer centres--leukaemia and relapsed malignancies are independent risk factors. *Eur. J. Pediatr.* **174**, 675 (May, 2015).
5. C. Bettegowda *et al.*, Detection of circulating tumor DNA in early- and late-stage human malignancies. *Sci. Transl. Med.* **6**, 224ra24 (Feb 19, 2014).
6. I. De Vlaminck *et al.*, Temporal response of the human virome to immunosuppression and antiviral therapy. *Cell* **155**, 1178 (Nov 21, 2013).
7. I. De Vlaminck *et al.*, Noninvasive monitoring of infection and rejection after lung transplantation. *Proc. Natl. Acad. Sci. U. S. A.* **112**, 13336 (Oct 27, 2015).
8. L. Epstein, I. See, J. R. Edwards, S. S. Magill, N. D. Thompson, Mucosal Barrier Injury Laboratory-Confirmed Bloodstream Infections (MBI-LCBI): Descriptive Analysis of Data Reported to National Healthcare Safety Network (NHSN), 2013. *Infect. Control Hosp. Epidemiol.* **37**, 2 (Jan, 2016).
9. A. Flagg, S. Worley, C. B. Foster, Characteristics of bacteremia in pediatric oncology patients based on pathogen classification as associated with the gastrointestinal mucosa or skin. *Infect. Control Hosp. Epidemiol.* **36**, 730 (Jun, 2015).
10. P. M. Flynn, B. Willis, A. H. Gaur, J. L. Shenep, Catheter design influences recurrence of catheter-related bloodstream infection in children with cancer. *J. Clin. Oncol.* **21**, 3520 (Sep 15, 2003).
11. G. Fratino *et al.*, Central venous catheter-related complications in children with oncological/hematological diseases: an observational study of 418 devices. *Ann. Oncol.* **16**, 648 (Apr, 2005).
12. A. Gafter-Gvili *et al.*, Antibiotic prophylaxis for bacterial infections in afebrile neutropenic patients following chemotherapy. *Cochrane Database Syst Rev* **1**, CD004386 (2012).
13. A. Goudie, L. Dynan, P. W. Brady, M. Rettiganti, Attributable cost and length of stay for central line-associated bloodstream infections. *Pediatrics* **133**, e1525 (Jun, 2014).
14. P. Gyarmati *et al.*, Metagenomic analysis of bloodstream infections in patients with acute leukemia and therapy-induced neutropenia. *Sci. Rep.* **6**, 23532 (Mar 21, 2016).

St. Jude Children's Research Hospital
IRB NUMBER: Pro00007383
IRB APPROVAL DATE: 07/29/2020

15. K. A. Hale *et al.*, Epidemiology of paediatric invasive fungal infections and a case-control study of risk factors in acute leukaemia or post stem cell transplant. *Br. J. Haematol.* **149**, 263 (Apr, 2010).
16. K. J. Henrickson *et al.*, Prevention of central venous catheter-related infections and thrombotic events in immunocompromised children by the use of vancomycin/ciprofloxacin/heparin flush solution: A randomized, multicenter, double-blind trial. *J. Clin. Oncol.* **18**, 1269 (Mar, 2000).
17. N. Hijiya *et al.*, Phase 2 trial of clofarabine in combination with etoposide and cyclophosphamide in pediatric patients with refractory or relapsed acute lymphoblastic leukemia. *Blood* **118**, 6043 (Dec 01, 2011).
18. D. K. Hong, M. Kertesz, T. Blauwkamp, C. Truong, N. Banaei, paper presented at the IDWeek 2016, New Orleans, October, 2016 2016.
19. J. Hord, J. Lawlor, N. H. O. C. C. , paper presented at the American Society of Pediatric Hematology/Oncology 24th Annual Meeting, Baltimore, Maryland, April 13-16, 2011 2011.
20. H. Inaba *et al.*, Infection-related complications during treatment for childhood acute lymphoblastic leukemia. *Ann. Oncol.* **28**, 386 (ePub ahead of print October 25, 2016, 2017).
21. S. Jeha *et al.*, Phase II study of clofarabine in pediatric patients with refractory or relapsed acute myeloid leukemia. *J. Clin. Oncol.* **27**, 4392 (Sep 10, 2009).
22. H. P. Lai *et al.*, Bacteremia in hematological and oncological children with febrile neutropenia: experience in a tertiary medical center in Taiwan. *J. Microbiol. Immunol. Infect.* **36**, 197 (Sep, 2003).
23. S. E. Lawson *et al.*, The UK experience in treating relapsed childhood acute lymphoblastic leukaemia: a report on the medical research council UKALLR1 study. *Br. J. Haematol.* **108**, 531 (Mar, 2000).
24. T. Leblanc *et al.*, Treatment of relapsed or refractory acute leukemia in childhood with bisantrene combined with high dose aracytine. *Med. Pediatr. Oncol.* **22**, 119 (1994).
25. M. Y. Lin, R. A. Weinstein, B. Hota, Delay of active antimicrobial therapy and mortality among patients with bacteremia: impact of severe neutropenia. *Antimicrob. Agents Chemother.* **52**, 3188 (Sep, 2008).
26. T. P. Lodise, Jr. *et al.*, Predictors of 30-day mortality among patients with Pseudomonas aeruginosa bloodstream infections: impact of delayed appropriate antibiotic selection. *Antimicrob. Agents Chemother.* **51**, 3510 (Oct, 2007).
27. A. J. McCarthy, L. A. Pitcher, I. M. Hann, A. Oakhill, FLAG (fludarabine, high-dose cytarabine, and G-CSF) for refractory and high-risk relapsed acute leukemia in children. *Med. Pediatr. Oncol.* **32**, 411 (Jun, 1999).
28. Y. H. Messinger *et al.*, Bortezomib with chemotherapy is highly active in advanced B-precursor acute lymphoblastic leukemia: Therapeutic Advances in Childhood Leukemia & Lymphoma (TACL) Study. *Blood* **120**, 285 (Jul 12, 2012).
29. B. J. Morland, P. J. Shaw, Induction toxicity of a modified Memorial Sloan-Kettering-New York II Protocol in children with relapsed acute lymphoblastic leukemia: a single institution study. *Med. Pediatr. Oncol.* **27**, 139 (Sep, 1996).

St. Jude Children's Research Hospital
IRB NUMBER: Pro00007383
IRB APPROVAL DATE: 07/29/2020

30. S. N. Naccache, A. Greninger, E. Samayoa, S. Miller, C. Y. Chiu, Clinical Utility of Unbiased Metagenomic Next-Generation Sequencing in Diagnosis of Acute Infectious Diseases: A Prospective Case Series. *Open forum infectious diseases* **2**, 103 (Dec, 2015).
31. S. N. Naccache *et al.*, Diagnosis of neuroinvasive astrovirus infection in an immunocompromised adult with encephalitis by unbiased next-generation sequencing. *Clin. Infect. Dis.* **60**, 919 (Mar 15, 2015).
32. M. J. Nielsen *et al.*, Viridans Group Streptococcal Infections in Children After Chemotherapy or Stem Cell Transplantation: A 10-year Review From a Tertiary Pediatric Hospital. *Medicine (Baltimore)* **95**, e2952 (Mar, 2016).
33. J. Ochs *et al.*, Teniposide (VM-26) and continuous infusion cytosine arabinoside for initial induction failure in childhood acute lymphoblastic leukemia. A Pediatric Oncology Group pilot study. *Cancer* **66**, 1671 (Oct 15, 1990).
34. I. Raad *et al.*, Ultrastructural analysis of indwelling vascular catheters: a quantitative relationship between luminal colonization and duration of placement. *J. Infect. Dis.* **168**, 400 (Aug, 1993).
35. R. Simon, Optimal two-stage designs for phase II clinical trials. *Control. Clin. Trials* **10**, 1 (Mar, 1989).
36. S. C. Stamou *et al.*, Hickman-Broviac catheter-related infections in children with malignancies. *Mt. Sinai J. Med.* **66**, 320 (Oct-Nov, 1999).
37. S. A. Sterling, W. R. Miller, J. Pryor, M. A. Puskarich, A. E. Jones, The Impact of Timing of Antibiotics on Outcomes in Severe Sepsis and Septic Shock: A Systematic Review and Meta-Analysis. *Crit. Care Med.* **43**, 1907 (Sep, 2015).
38. B. Thomson *et al.*, Toxicity and efficacy of intensive chemotherapy for children with acute lymphoblastic leukemia (ALL) after first bone marrow or extramedullary relapse. *Pediatr. Blood Cancer* **43**, 571 (Oct, 2004).
39. P. Trioche *et al.*, French "real life" experience of clofarabine in children with refractory or relapsed acute lymphoblastic leukaemia. *Experimental hematology & oncology* **1**, 39 (Dec 10, 2012).
40. H. van den Berg *et al.*, Outcome after first relapse in children with acute lymphoblastic leukemia: a report based on the Dutch Childhood Oncology Group (DCOG) relapse all 98 protocol. *Pediatr. Blood Cancer* **57**, 210 (Aug, 2011).
41. R. J. Wells *et al.*, Mitoxantrone and cytarabine induction, high-dose cytarabine, and etoposide intensification for pediatric patients with relapsed or refractory acute myeloid leukemia: Children's Cancer Group Study 2951. *J. Clin. Oncol.* **21**, 2940 (Aug 01, 2003).
42. R. J. Wells *et al.*, Cytosine arabinoside and mitoxantrone treatment of relapsed or refractory childhood leukemia: initial response and relationship to multidrug resistance gene 1. *Med. Pediatr. Oncol.* **22**, 244 (1994).
43. J. A. Whitlock *et al.*, High-dose cytosine arabinoside and etoposide: an effective regimen without anthracyclines for refractory childhood acute non-lymphocytic leukemia. *Leukemia* **11**, 185 (Feb, 1997).
44. M. Z. Wilson, C. Rafferty, D. Deeter, M. A. Comito, C. S. Hollenbeak, Attributable costs of central line-associated bloodstream infections in a pediatric hematology/oncology population. *Am. J. Infect. Control* **42**, 1157 (Nov, 2014).

St. Jude Children's Research Hospital
IRB NUMBER: Pro00007383
IRB APPROVAL DATE: 07/29/2020

45. E. Zimlichman *et al.*, Health Care-Associated Infections: A Meta-analysis of Costs and Financial Impact on the US Health Care System. *JAMA Intern Med* **173**, 2039 (Dec 9, 2013).

St. Jude Children's Research Hospital
IRB NUMBER: Pro00007383
IRB APPROVAL DATE: 07/29/2020

APPENDICES

APPENDIX I: SCHEDULE OF EVALUATIONS

Event	Study Entry	During study (Weekly)	Collection of final sample +7 days
Data collection			
Demographic data	X		
Past history (30 days)	X		
Infectious episodes		X	X
Antibiotic exposure		X	X
Sample collection			
Plasma samples		X[a]	

[a]Up to daily when leftover samples available.

St. Jude Children's Research Hospital
IRB NUMBER: Pro00007383
IRB APPROVAL DATE: 07/29/2020

APPENDIX II: RESEARCH TESTS

**Research Test**	**Course**	
Next generation plasma pathogen sequencing	Prior to and during each infectious episode	Required
Cell free DNA disease monitoring	Prior to and during each cancer-related episode	Required

St. Jude Children's Research Hospital
IRB NUMBER: Pro00007383
IRB APPROVAL DATE: 07/29/2020

APPENDIX IV: SAMPLE PROCESSING

A running list of participants in the study will be maintained by the study team.

To collect clinical samples, a member of the study team will go to the clinical lab to collect available samples for participants that were collected in EDTA or other heparin-free tubes.

The samples will be taken to the Tuomanen/Rosch/Margolis lab complex where they will first be centrifuged at 1,600 x g for 10 minutes. The plasma will then be transferred to a second tube which will be spun at 16,000 x g for another ten minutes. The supernatant will be removed and transferred in 750ul aliquots to new microcentrifuge tubes.

The samples will be deidentified at that time prior to placing a coded label. The samples will be stored in a -80 freezer prior to shipping to Karius. Information on samples being included in the study will be given in batches of a minimum of ten samples to make sure those acquiring outcome data will remain blinded.

Samples for NGS analysis will be transferred to Karius inc. for testing. One 750ul aliquot of each sample will be sent to:

David Hong, MD
Karius Inc.
1505A Adams Drive
Menlo Park, CA 94025

Karius will perform nucleic acid isolation, sequencing, and data analysis using their CLIA-approved clinical pipeline. They will provide a list of microbes identified in each sample to the investigators.

Aliquots of plasma not used in the study may undergo standard cell-free DNA isolation, library preparation, exome capture, sequencing, and analysis.After completion of all study procedures and assessments, any unused samples may be placed in an institutional sample bank for use in future IRB approved studies.

St. Jude Children's Research Hospital
IRB NUMBER: Pro00007383
IRB APPROVAL DATE: 07/29/2020
